# Selection of seismic isolation system parameters for the near-optimal design of structures

**DOI:** 10.1038/s41598-022-19114-7

**Published:** 2022-08-30

**Authors:** Moussa Leblouba

**Affiliations:** Department of Civil & Environmental Engineering, College of Engineering, University of Sharjah, P.O.BOX 27272, Sharjah, UAE

**Keywords:** Civil engineering, Mechanical engineering

## Abstract

The benefits of seismic isolation are many. Structures that are isolated from the ground seismically perform better than those that are not. They experience reduced floor accelerations and drifts and are less likely to experience damage to structural elements. Additionally, their contents are better protected from the effects of earthquakes. The selection and design of seismic isolation devices are complex and require a good understanding of how they behave during earthquakes. This study investigates the effect of various isolation system parameters and ground motion characteristics on the seismic response of base isolated structures in order to develop rational procedures for design and analysis. Additionally, the study investigates the problem of optimal design of seismic isolation systems through parametric nonlinear dynamic analysis. Results showed that the maximum base shear and displacement were velocity-sensitive and that the peak ground velocity controls the motion. The largest maximum base shear occurred when using isolation systems with high yield strength levels and low degrees of nonlinearity, while the smallest maximum base shear occurred when using low yield strength levels and high degrees of nonlinearity. Results from the study can be used to select the appropriate isolation devices and design them correctly to achieve the benefits they provide.

## Introduction

Structural protective add-on hardware developed to protect structures subjected to earthquakes^1,2^ are grouped into three broad areas, base isolation, passive energy dissipation, and active control. Passive control devices have been successfully used to reduce the dynamic response of structures subjected to severe earthquakes; their first use began since the 1970s. Energy dissipating devices can be classified into three categories^3^: viscous and viscoelastic dampers, metallic dampers, and friction dampers.

Seismic isolation is relatively a new technique of seismic resistant design of buildings, bridge structures, and nuclear power plants^4^. However, the idea of vibration isolation existed from the beginning of the $20^{th}$ century; after different stages and developments, particularly within the last 30 years, this technique has become a practical reality with the invention of different seismic isolation devices.

The principle of seismic isolation is to provide discontinuity between two bodies in contact so that the motion of either body in the direction of the discontinuity cannot be fully transmitted; this results in a significant reduction in floor acceleration and interstory drifts. Hence, protecting to the building’s precious contents and components. For its great performance, in the USA, Japan, Italy, and New Zealand. The technique of seismic isolation has now advanced to the point where it is often considered for the protection of both new and existing buildings^5^.

Contemporary buildings contain high costly equipment and contents that must be protected against earthquakes and be operational after a severe ground shaking; such buildings are those designated for research, health care, telecommunication, nuclear power plants, etc. Buildings constructed following the old seismic codes, with conventional resistant design approaches such as shear walls, braced frames, and moment-resistant frames, cannot protect the valuable equipment that contain these buildings.

Seismic isolation is not intended to enhance the capacity of a building but rather is considered a means of reducing the seismic demand on the structure.

Most of the existing buildings and bridges use elastomeric bearings, with the elastomer being either natural rubber or neoprene or sliding bearings, with the sliding surface being Teflon and stainless steel. Hence, isolation systems may be divided into two categories; the first category includes the family of elastomeric bearings^6,7^, in which we find the high damping rubber bearing system (HDRB), the lead rubber bearing system (LRBs) and other systems. The second category includes the family of sliding bearings, in which we find the friction pendulum system (FPS)^8^ and sliding bearing^9^ with and without system without recentering (SI), among others^10^.

The lateral stiffness of an isolator is extremely small when compared to its vertical stiffness, and an isolator is almost elastic for lateral deformations within its radius. There are even devices that have negative stiffness^11,12^. The isolation system does not absorb the earthquake energy but rather deflects it through the dynamics of the isolation system.

Several factors influence the selection and design of isolation devices. The selection of the appropriate isolation device is based on some requirements ranging from lateral and vertical stiffness, and cost benefits to the durability, the design of the isolation system is based on several requirements and policies. Several design methods were proposed, some based on elastic spectra, others based on linearized behavior of base isolated structures, and others proposed to be included in seismic codes.

Experimental^13–16^, numerical and analytical studies^17–19^ performed on single isolation devices showed that their important characteristics (horizontal and vertical stiffness, strength, etc.) depend on load cases, and demonstrated that axial loads have a significant effect when combined with lateral loads. For instance, Kalpakidis et al.^20^ showed that the strength of lead rubber bearings degrades when subjected to large deformations. The authors attributed the strength degradation to the heating of the lead core and then proposed a method to incorporate strength degradation due to lead core heating in modeling the hysteretic behavior of lead–rubber bearings. Elastomeric bearings have also been shown to fail under large tensile strains due to the formation of cavities and rupture. Kumar et al.^21^ conducted a series of tests on 16 low-damping rubber bearings to investigate the effect of cavitation on the shear and axial properties of elastomeric bearings. The authors developed and validated a phenomenological model of elastomeric bearings in tension.

## Problem statement and research objectives

This study was motivated by the need to investigate the performance of base isolated structures during earthquake ground motions. Some existing design procedures^22^ and design guidelines require many parameters to be calculated and a long path to be followed to achieve to the required base isolation system to be used for a building. This constitutes another motivation to think about another approach in the design that can help engineers in the preliminary design process to select the near-optimal isolation device parameters that can later be checked using nonlinear time history analysis.

Some of the proposed design procedures^23^ are complex and less reliable, while others are based on linear theory and are less accurate since isolation systems are inherently nonlinear especially lead-rubber bearing system and friction pendulum system. To account for the inherent uncertainty in the design parameters of isolation systems, reliability-based design methods have been proposed. For instance, Castaldo et al.^24–26^ developed seismic reliability-based ductility demand analysis techniques for base-isolated structures with friction pendulum systems.

Seismic isolation systems have demonstrated their effectiveness over multiple structures that incorporated them during recent earthquakes or tests^27^, but other base isolated structures did not perform as expected. FCLJC (Foothill Communities Law and Justice Center, USA) is an example of these structures, which did not act under the Loma Prieta recent earthquake (October 1989 and February 1990), Kelly et *al.*^28^ explained the response of the FCLJC by the fact that effective isolation period at the level of deformation induced in the elastomer is about the same as that of the fixed-base superstructure, hence, the structure did perform with smaller drifts and smaller forces than would the fixed-base structure subjected to the same input motion.

The main drawbacks of seismic isolation systems are large lateral base deflections and undesirable motion under wind and other minor excitations. To prevent excessive lateral deformation under small earthquakes and dissipate energy under strong earthquakes, special devices can be used and attached at the foundation level and any other desired floor level. Experiments conducted by Kelly et al. on a half-scale model of a steel framed structure employing energy absorbing devices attached at the bottom floor girders show that for small input motion, the structure behaved as if attached to a rigid foundation, strongly amplifying the ground motion. In contrast for strong earthquakes, the devices yielded and absorbed large amounts of energy amounting to as much as the equivalent of 30%-35% of critical damping.

Bhatti et al.^29^ performed an optimization analysis to get the best device parameters of the energy absorbing devices through nonlinear programming techniques, but their study was for a particular structure. Jangid^30^ conducted a parametric study of base-isolated structures with different isolation systems, he found that the damping and the period of the superstructure do not have noticeable effects on the peak response of base-isolated structures, and the effects of viscous damping have little influence when the additional damping in the isolation system in the form of such as hysteretic is present.

This study investigates the problem of optimal design of seismic base isolation systems through parametric nonlinear dynamic analysis. To that end, the main objectives of this study are as follows:

- To review the seismic isolation technology and different types of isolation devices.
- To analyze the behavior and performance of a typical base isolated building.
- To conduct a parametric analysis to determine the influence of every variable on selected response parameters of interest and to search for the near-optimal seismic isolation design parameters for a selected suite of ground motions.

## Review of related studies

Earthquake imparts to the structure a great amount of energy that causes damage to structural elements as well as to its equipment. Conventional seismic resistant design strategies that insert reinforced concrete walls, bracing, or other traditional systems to resist earthquakes did not protect structures against severe ground motions. The concept of seismic isolation is a relatively new technique in earthquake engineering; its principle is to provide a discontinuity between the foundation and the superstructure so that the seismic energy cannot be fully transmitted into the structure, this results in a significant reduction in floor acceleration and interstory drifts^5^.

The conventional earthquake resistant design relies on the strength and ductility of the structural components to resist earthquake-induced forces and dissipate the seismic energy, thereby preventing the collapse of structures in case of an earthquake. In contrast, base isolation approach aims to reduce the damaging horizontal seismic force transmitted to the structures^31^.

Seismic isolation is not intended to enhance the capacity of a building but rather is considered a means of reducing the seismic demand on the structure. In recent years base isolation has become an increasingly applied structural design technique for buildings and bridges in highly seismic areas. Many structures have been built using this approach, and many others are in the design phase or under construction.

A practical seismic isolation system should meet the following three requirements^32^:

- Sufficient horizontal flexibility to increase the structural period and spectral demands, except for very soft soil sites;
- Sufficient energy dissipation capacity to limit the displacements across the isolators to a practical level;
- Adequate rigidity to make the isolated building not different from a fixed-base building under general service loading.

In addition, after Kelly et al.^33^ the basic requirements of a base isolation system for earthquake protection are that :

- The bearings must support the dead load of the structure and must have a high vertical stiffness;
- The horizontal stiffness of the bearings must provide a horizontal natural frequency low enough that the building will not respond to the destructive components of the ground motion. From the response spectra of Seed it is clear that under a wide range of conditions a horizontal natural frequency of 0 5Hz is appropriate;
- Some earthquake energy will always occur at or near the horizontal natural frequency so the system must contain sufficient damping to limit translational movement to an acceptable level;
- The isolation bearing system must prevent excessive movement of the building under wind loading. it must be evident that this requirement is not on the grounds of safety-the system is designed to prevent damage due to much more severe effects-but primarily for the comfort of the occupants as even a slight swaying motion might be disconcerting.

Most commonly used seismic isolating systems can satisfy all the above requirements. Certainly, if the seismic isolating system can be equipped with fail-safe devices to avoid the total collapse of the isolated structure in the case where excessive displacements occur, then the system will most likely be satisfactory.

The first period of an R/C building is about $0.02\;H\;sec.$ (*H*: height, *m*). Accordingly, the first period of a 15 meter high building is about 0.3 of a second. This period could easily be extended to 3 seconds by supporting the building with isolators. A period shift from 0.3 to 3 seconds provides a different seismic force into the building. A different seismic force primarily depends on the period and amplitude characteristics of ground motion^34^.

**Figure 1:**
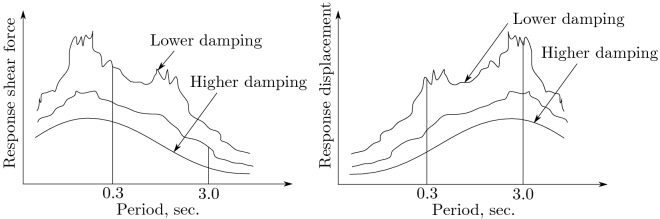
Ideal response curve.

Therefore, if the ground motion includes many components whose periods are close to 3.0 seconds, such components will amplify the building motion, but others will not. Seismic features change the response of buildings; the general relationship between ground motion and the response is indicated in Fig. 1. The figure shows large differences in seismic force between “a building coupled to the ground rigidly (example: period $0.3\;sec.$)” and “a building supported by isolators (example: period $3\;sec.$)”. This also indicates that the damping controls the response.

Ideally, the isolators should be located as low as possible in a structure to protect as much of the structure as possible. However, cost and practical considerations influence the choice of location. In a building, the choice may lie between isolating at ground level, below the basement, or at some point up the columns. Each location has advantages and disadvantages relating to accessibility and other very important design considerations such as cladding, partitions, and building services^35^.

Seismic isolators come in different forms, ranging from infinitely thin sliding surfaces (bearings) to multiple layers of rubber a few centimeters thick mounted at the base, the flexible or absorbing structural members of any depth. Because vertical stiffness is generally required for most gravity loads, seismic isolation is only appropriate for horizontal motions^35^.

Generally, two categories of isolation systems exist and are widely used. The first category includes the family of elastomeric bearings, in which we find the high damping rubber bearing system (HDRB), the lead rubber bearing system (LRBs), etc. According to Kelly^36^, in this category, the building or structure is decoupled from the horizontal components of the earthquake ground motion by interposing a layer with low horizontal stiffness between the structure and the foundation. This layer gives the structure a fundamental frequency much lower than its fixed-base frequency and much lower than the predominant frequencies of the ground motion. The first dynamic mode of the isolated structure involves deformation only in the isolation system, the structure above being rigid. The higher modes that will produce deformation in the structure are orthogonal to the first mode and the ground motion. These higher modes do not participate in the motion so if there is high energy in the ground motion at these higher frequencies, this energy cannot be transmitted into the structure. The isolation system does not absorb the earthquake energy but rather deflects it through the dynamics of the system. This type of isolation works when the system is linear and even when undamped; however, some damping is beneficial to suppress any possible resonance at the isolation frequency^36^.

The second category includes the family of sliding bearings, in which we find the friction pendulum system (FPS) and sliding bearing system without re-centering (SI)^32^. The isolator of this category works by limiting the transfer of shear across the isolation interface. Many sliding systems have been proposed, and some have been used. In China, at least three buildings on sliding systems use specially selected sand at the sliding interface. A type of isolation containing a lead-bronze plate sliding on stainless steel with an elastomeric bearing has been used for a nuclear power plant in South Africa. The friction-pendulum system is a sliding system using a special interfacial material sliding on stainless steel and has been used for several projects in the United States, both new and retrofit construction^31,33,36,37^.

Conceptually, any of the isolation systems that exist are required to fulfill the following performance objectives:

- Flexibility;
- Damping;
- Resistance to service loads.

## Modeling of base isolated buildings

The basic concept of base isolation design is to minimize the earthquake force transmitted into a superstructure while simultaneously suppressing the deformation of isolators within an allowable range. A superstructure is expected to absorb significantly less energy when compared to the base isolation level. The seismic capacity of a superstructure will correspond to the response of a superstructure regarded as a rigid body. Thus, the superstructure can be released from several conditions which have restricted conventional buildings from having sufficient energy absorbing capacity (i.e., ductility).

By carefully selecting and locating isolation devices, the superstructure can be released from the harmful influence of torsional vibration caused by an eccentricity between the center of mass and the center of rigidity in the superstructure. Therefore, a base isolation system can provide a much more flexible and simple design procedure than a conventional system.

The conventional seismic design has provided extra bearing capacity for load, except for seismic loads. Actually, this load is the main design load of a base isolated building. This load should then be estimated precisely in the preliminary design.

Different levels of modeling can be set up for a base isolation structure, from ones that are simple single-mass models to those which are complicated 3D models. Time history analysis using these models is an efficient way to obtain the actual response. Several different levels of response analysis models can be established according to the purposes involved, from simple to more complicated^38^. No matter what level of complexity is the mathematical model, in general the superstructure is generally modeled as a linear shear system with the nonlinearity concentrated at the base isolation level. The response of each floor level can thereby be obtained^34^ after a nonlinear time history analysis.

### Isolation system modeling

The isolation system is considered to be nonlinear. The nonlinear force-displacement characteristics of the isolation components are modeled explicitly using spring elements or a combination of springs and dashpots. The models for isolation components are described here. The following assumption is made for modeling the isolation system^38^:

- The isolation system is rigid in the vertical direction and torque resistance of individual bearing is neglected.
- The differential equation model for the uniaxial behavior developed by Wen^39^ and the differential equation model for the biaxial behavior developed by Park et al.^40^.
- The essential features that need to be modeled for uniaxial behavior of elastomeric bearings are the appropriate shear stiffness representation in the pre-and-post yielding range, representation of the strain dependence of shear stiffness because of P-Delta effects.

### Models for isolation systems

There are many isolation elements that can be considered for isolation system modeling; there are elastic, viscous, hysteretic elements for bilinear elastomeric bearings and hysteretic elements for sliding bearings. The hysteretic elements can be uniaxial or biaxial, and the linear elastic and viscous elements are considered for modeling linear elastomeric bearings and fluid dampers. The biaxial hysteretic behavior of bilinear elastomeric bearings and for frictional bearings is modeled using the biaxial interaction equations of Bouc-Wen model^39^.

**Figure 2:**
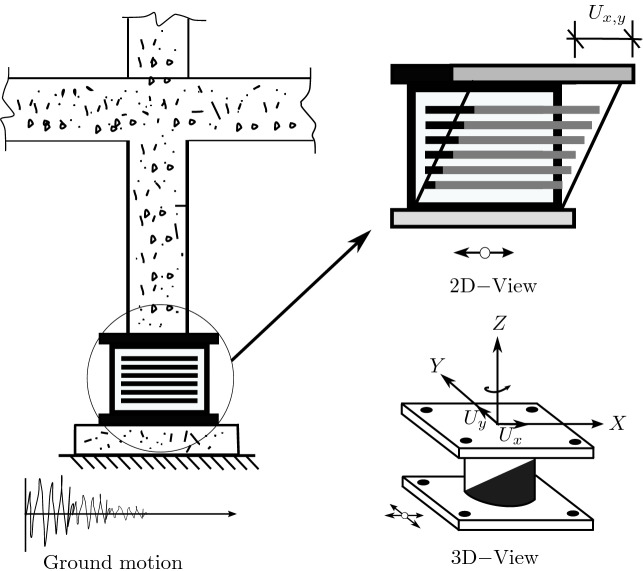
A bearing under column excited by an earthquake ground motion.

Consider a bearing under a column of a building, when the structure is subjected to a strong earthquake, the bearing displaces with displacement components $U_x$ and $U_y$(see Fig. 2).

A torsional moment develops at the bearing, but the contribution of this torsional moment to the total torque extend to the structure supported by several bearings is insignificant, so it will be neglected in the modeling. In addition, lateral forces develop and exhibit biaxial interaction, naturally these forces are opposite to direction of the ground motion.

The direction of the mobilized force *F* in the elastomeric bearings given by:

1 $$\begin{aligned} \theta =tan^-1\left( \frac{\dot{U_x}}{\dot{U_y}}\right) \;\;\;\;\;\;\;\;\;\; \dot{U}=\sqrt{\dot{U^{2}_{x}}+\dot{U^{2}_{y}}} \end{aligned}$$

**Figure 3:**
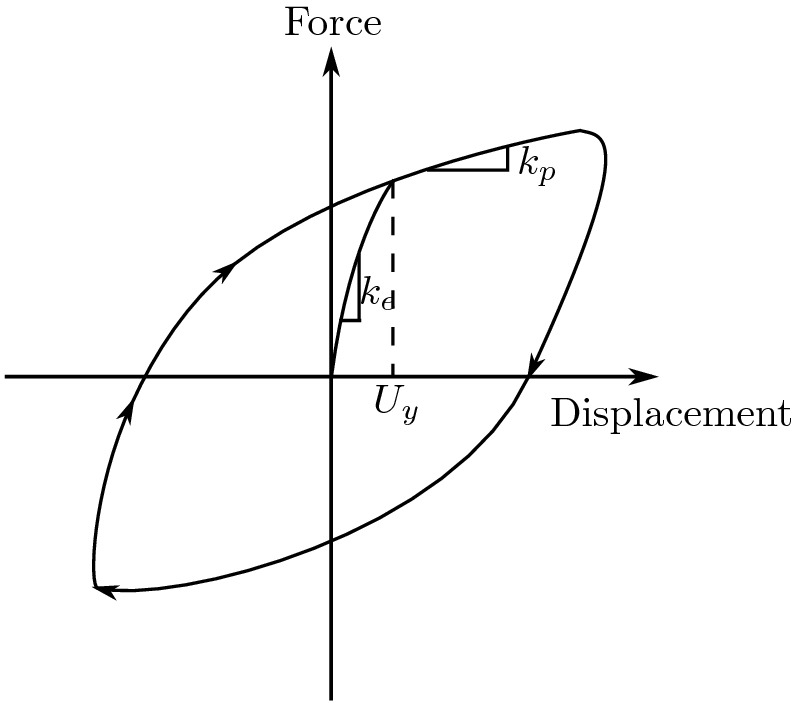
The force-displacement characteristics of the bearing.

The force-displacement characteristics of the bearing are shown in Fig. 3.

Now, we can write the equations using the Bouc–Wen model of hysreresis^38,39^:

2 $$\begin{aligned}&U^y\times \left\{ \begin{array}{l l} \dot{z}_x\\ \dot{z}_y \end{array} \right\} =\alpha \left\{ \begin{array}{l l} \dot{U}_x \\ \dot{U}_y \end{array} \right\} - Z_w\left\{ \begin{array}{l l} \dot{U}_x \\ \dot{U}_y \end{array}\right\} \end{aligned}$$

3 $$ z_{w}  = \left[ {\begin{array}{*{20}c}    {Z_{x}^{2} (\gamma \;{\text{ sign }}(\mathop {U_{x} }\limits^{.} Z_{x}  + \beta )} & {amp;Z_{x} Z_{y} (\gamma \;{\text{ sign }}(\mathop {U_{y} }\limits^{.} Z_{y}  + \beta )}  \\    {Z_{x} Z_{y} (\gamma \;{\text{ sign }}(\mathop {U_{y} }\limits^{.} Z_{x}  + \beta )} & {amp;Z_{y}^{2} (\gamma \;{\text{ sign }}(\mathop {U_{y} }\limits^{.} Z_{y}  + \beta )}  \\   \end{array} } \right] $$

where: $$Z_{x}$$, $$ Z_{y}$$ are dimensionless hysteretic variables that are bounded by values $$\pm 1$$; $$\alpha ,\; \beta ,\; \gamma $$ dimensionless quantities that control the shape of the hysteretic loop; $$\dot{U_{x}},\;\dot{U_{y}}$$ are the velocities in the *X* and *Y* directions, respectively; $$U^y$$ is the yield displacement.

When yielding begins, Eq. (3) leads to $Z_{x} = \cos {(\theta )}$ and $Z_{y} =\sin {(\theta }$ (considering $\alpha =1$, $\beta =0.1$, and $\gamma =0.9$, meaning $\alpha /(\beta +\gamma )$=1 as recommended by^38^.

The biaxial interaction can be neglected when the off-diagonal terms of the matrix in Eq. (3) are replaced by zeros. This results in uniaxial model with two independent elements in two orthogonal directions.

**Figure 4:**
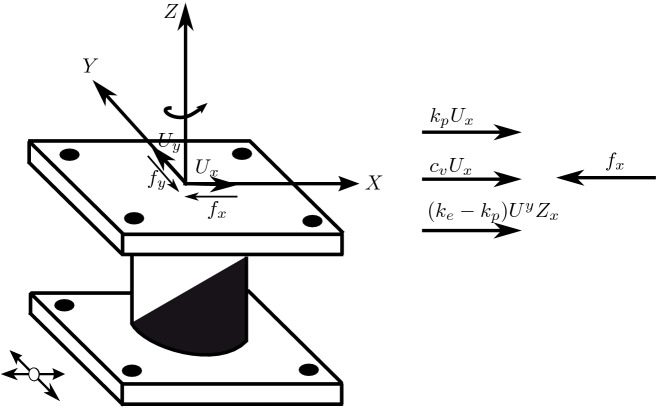
Forces equilibrium at the bearing.

Now, the forces mobilized in the elastomeric bearings as shown in Fig. 4 can be modeled by an elastic-viscoplastic model with strain hardening. The equations that characterize the mobilized forces are given by:

4 $$\begin{aligned} f_{x}=  k_{p}U_{x}+c_{v}\dot{U_{x}}+(k_{e}-k_{p})U^{y}z_{x} \end{aligned}$$

5 $$\begin{aligned} f_{y}= k_{p}U_{y}+c_{v}\dot{U_{y}}+(k_{e}-k_{p})U^{y}z_{y} \end{aligned}$$

in which $k_{e}$ is pre-yield stiffness; $k_{p}$ is the post-yield stiffness; $c_{v}$ is the viscous damping coefficient of the elastomeric bearing or device. Note that Eq. (3) can also be used to model sliding bearings with flat or spherical sliding surfaces by means of a small yield displacement $U^{y}$ (because of rigid plastic behavior and large stiffness) and setting $c_{v}$ =0 and $(k_{e}-k_{p})U^{y}$=$\mu N$. Here, $\mu$ is coefficient of friction and *N* represents the average normal force at the bearing (normal force variation is neglected).

**Figure 5:**
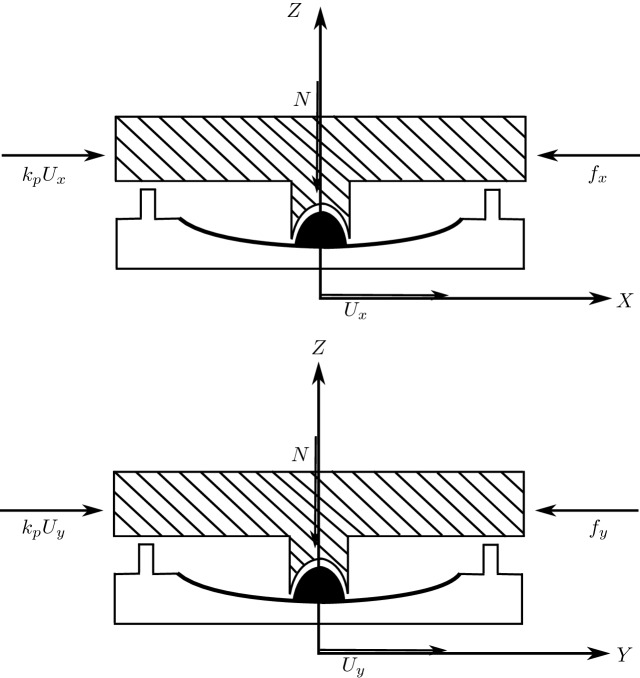
Forces equilibrium at the pendulum bearing.

**Figure 6:**
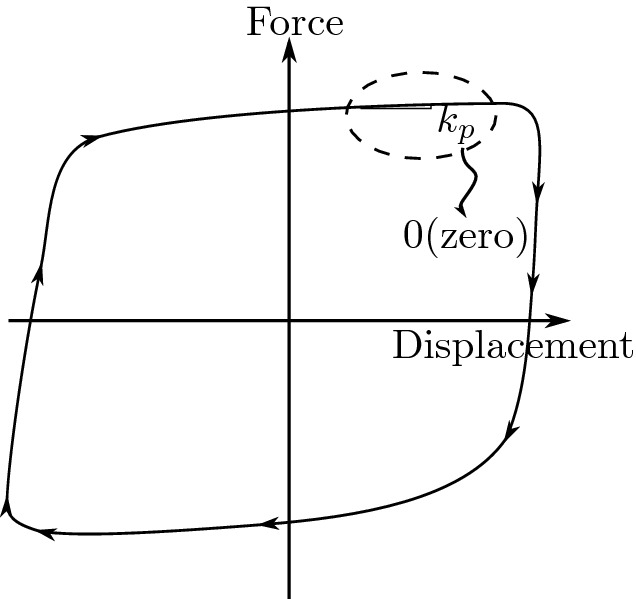
Negligible post-yield stiffness for FPS.

Figure 5 illustrate the forces equilibrium at the bearing. The forces mobilized in the bearing can be written as follows:

6 $$\begin{aligned} f_{x}= k_{p}U_{x}+\mu Nz_{x} \end{aligned}$$

7 $$\begin{aligned} f_{y}= k_{p}U_{y}+\mu Nz_{y} \end{aligned}$$

$k_{p}U_{x}$ and $k_{p}U_{y}$ represent the re-centering force due to the spherical surface of a friction pendulum bearing or a flat slider. Equation 6 can be simplified further considering $k_{p}$ as a negligible quantity (see Fig. 6), the forces mobilized in the bearing will be written as:

8 $$\begin{aligned} f_{x}=  \mu Nz_{x} \end{aligned}$$

9 $$\begin{aligned} f_{y}=  \mu Nz_{y} \end{aligned}$$

So we have arrived at the same equation found by Constantinou et al.^38^. Again, note that other isolation devices such as nonlinear fluid dampers can also be modeled using Eq. (3).

## Nonlinear dynamic modeling of base isolated structures

Base isolated buildings can be designed such that the superstructure remains elastic, also can be modeled by a condensed linear elastic system with concentrated nonlinearities at the isolation level. This technique allows the use of the Fast Nonlinear Analysis (FNA)^41^. Considering in addition, the base and the floors are infinitely rigid in plane (displacement in vertical are neglected), three degrees of freedom for each floor at the center of mass.

### Equations of motion of the fixed base structure

**Figure 7:**
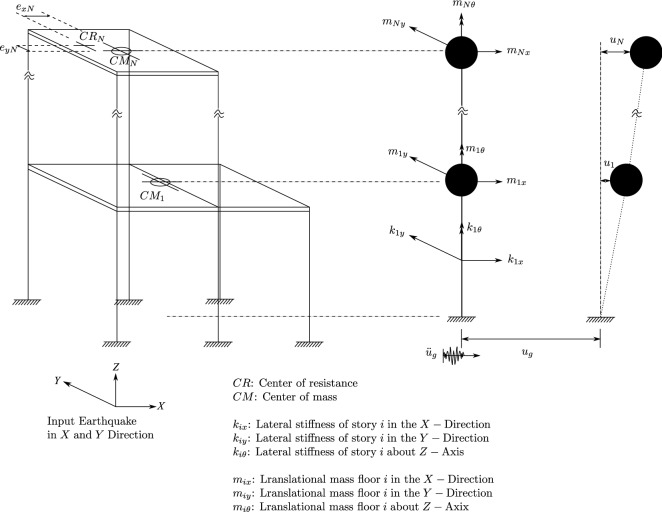
Model of a fixed base structure.

The equations of motion of the fixed base structure (Fig. 7) are as follows:

10 $$\begin{aligned} M\ddot{U}+C\dot{U}+KU=-MR\ddot{U_g} \end{aligned}$$

In which: *M*, *C*, *K* are the mass, damping, and stiffness matrices of the structure; *R* is the matrix of earthquake influence coefficients. By the mode superposition method, $U=\sum _{i}^N Y_{i}\phi _{i}$. The properties of orthogonality help us to write:

11 $$\begin{aligned}&\phi _{i}^{T}M\phi _{i}=0\;\;\;\;\;\;\phi _{i}^{T}K\phi _{j}=0 \;\;\;\;\;\;\text{ for }\;i\ne j,\;\text{ so, } \end{aligned}$$

12 $$\begin{aligned}&\phi ^{T}M\phi \ddot{Y(t)}+\phi ^{T}C\phi \dot{Y(t)}+\phi ^{T}K\phi Y(t)=-MR\ddot{U_{g}} \end{aligned}$$

The equations of motion can be re-written in the alternative form:

13 $$\begin{aligned}&\ddot{Y(t)}+2\xi \omega \dot{Y(t)}+\omega ^2Y(t)=-MR/M^*\ddot{u_{g}} \end{aligned}$$

14 $$\begin{aligned}&C^*=\phi ^{T}C\phi \;\; M^*=\phi ^{T}M\phi \end{aligned}$$

*C* matrix can be found using the Rayleigh damping:

15 $$\begin{aligned} C=a_{0}M+a_{1}K \left\{ \begin{array}{l l} a_0\\ a_1 \end{array} \right\} =\frac{2\xi }{\omega _1+\omega _{N}}\left\{ \begin{array}{l l} \omega _1\omega _{N}\\ 1 \end{array} \right\} \end{aligned}$$

in which $\omega _1$ is the first circylar frequency and $\omega _{N}$ is the higher preponderant frequency.

### Equations of motion of the isolated structure

**Figure 8:**
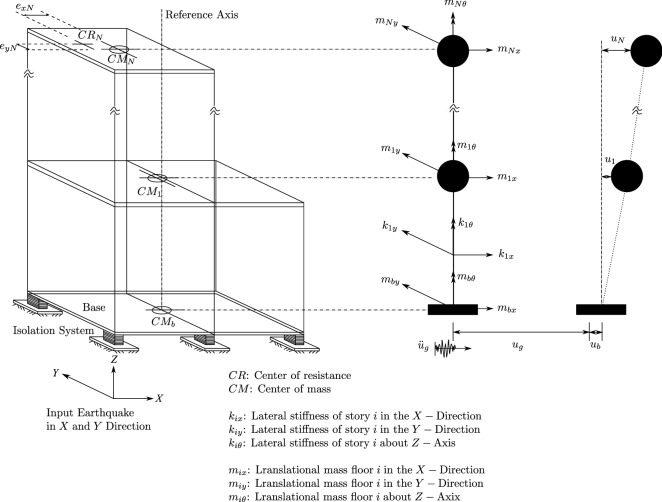
Model of a base isolated structure.

The equations of motion of a base isolated structure can be divided into two sets, one for the superstructure and one for the base. The equations of motion of the elastic superstructure are (Fig. 8):

16 $$\begin{aligned} M\ddot{U}+C\dot{U}+KU=-MR(\ddot{U_{g}}+\ddot{U_{b}}) \end{aligned}$$

The equations of motion of the base are:

17 $$\begin{aligned} R^{T}M\left[ \ddot{U}+R\left( \ddot{U_{g}}+\ddot{U_{b}}\right) \right] +M_{b}\left( \ddot{U_{g}}+\ddot{U_{b}}\right) +C_{b}\dot{U_b}+K_{b}U_{b}+f=0 \end{aligned}$$

in which, $M_{b}$, $C_{b}$, $K_{b}$ are, respectively, the diagonal mass, damping, and stiffness matrices of the rigid base; *f* is the vector containing the forces mobilized in the nonlinear elements of the isolation system.

Using the modal reduction technique:

18 $$\begin{aligned} U=\phi U^* \;\;\; or \;\;\;U=\phi _{n\times n} U_{m\times 1}^* \end{aligned}$$

In which $\phi$ is the model matrix and $U^*$ is the model displacement vector relative to the base and *m* is the number of eigenvectors retained in the analysis, Eq. (17) combined with Eq. (18) gives:

19 $$ \begin{gathered}   \left( {\begin{array}{*{20}c}    {\left[ I \right]} & {\left[ {\phi ^{T} MR} \right]}  \\    {\left[ {R^{T} M\phi } \right]} & {\left[ {R^{T} MR\phi  + M_{b} } \right]}  \\   \end{array} } \right)\left\{ {\begin{array}{*{20}l}    {\mathop {u^{*} }\limits^{{..}} } \hfill  \\    {\mathop {u_{b} }\limits^{{..}} } \hfill  \\   \end{array} } \right\} + \left( {\begin{array}{*{20}c}    {\left[ {2\xi _{i} } \right]} & 0  \\    0 & {\left[ {c_{b} } \right]}  \\   \end{array} } \right) +  \hfill \\   \quad \quad \quad \quad \quad \quad \quad \quad \quad \quad \quad \quad \quad \quad \quad \quad  + \left( {\begin{array}{*{20}c}    {\left[ {\omega _{i}^{2} } \right]} & 0  \\    0 & {\left[ {k_{b} } \right]}  \\   \end{array} } \right)\left\{ {\begin{array}{*{20}l}    {u^{*} } \hfill  \\    {u_{b} } \hfill  \\   \end{array} } \right\} + \left\{ {\begin{array}{*{20}l}    0 \hfill  \\    f \hfill  \\   \end{array} } \right\} =  - \left( {\begin{array}{*{20}c}    {\phi ^{T} MR}  \\    {R^{T} MR\phi  + M_{b} }  \\   \end{array} } \right){\text{ }} \hfill \\  \end{gathered}  $$

$\xi _{i}$ and $\omega _{i}$ are the damping ratio and the circular frequency for the fixed base structure in mode *i*. Note that, the matrices $\left[ 2\xi _{i}\right]$ and $\left[ \omega _{i}^2\right]$ are diagonal. Nagarajiah et al.^38^ used the pseudo-force method^42,43^ in their software 3D-BASIS^38^ to solve the above equations of motion. This was justified by the disadvantages proved in using traditional methods. For instance, the authors found that, unlike the pseudo-force method, the Newton-Raphson method didn’t converge to the solution in the case of severe nonlinearities like those present in sliding isolation systems.

In the pseudo-force method, the equations of motion are first written in an incremental form then the vector of nonlinear forces is brought to the right-hand side of the equations of motion and treated as a pseudo-force vector. The solution algorithm uses Newmark’s constant-average acceleration method, which is unconditionally stable even for negative tangent stiffness^44^. An iterative step is executed in each time step until equilibrium is achieved. The complete solution algorithm can be found in^38^.

A Matlab code was developed to solve Eq. (19) using the pseudo-force method. The code was used to analyze the dynamic response of a three-story base isolated reinforced concrete building subjected to El Centro 1940 ground motion (NS component). The isolation system consists of lead rubber bearing system characterized by a degree of nonlinearity of $1/\alpha = 13.5$ and a yield strength level of $\mu = 7\%$, designed to achieve an isolation period of vibration $T_b = 2.5$ seconds. Figure X shows the base shear-base displacement hysteresis curve.

**Figure 9:**
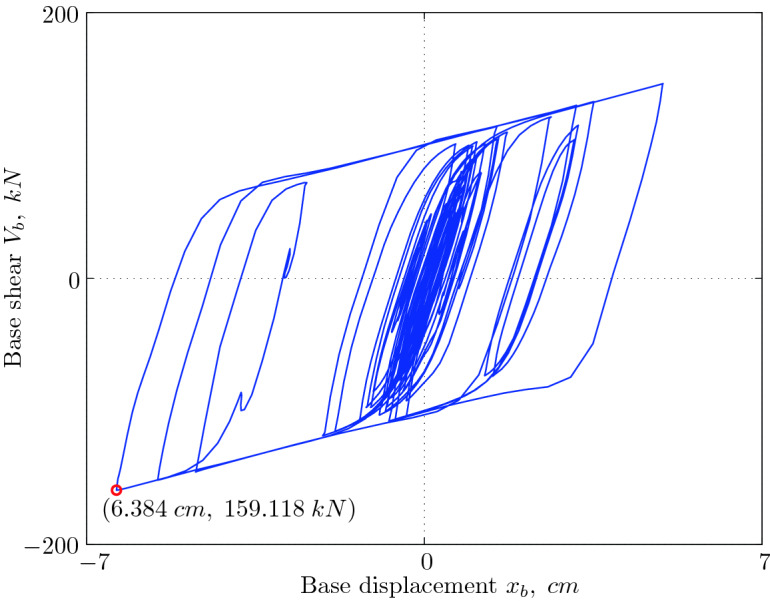
Base shear-base displacement hysteresis.

## Near optimal seismic isolation design parameters

To achieve to the last objectives of this study, in this section we search for the nearly best (i.e., near optimal) isolation system design parameters for structures through an extensive parametric analysis considering isolation system parameters and different earthquake ground motions.

### Analysis procedure

**Figure 10:**
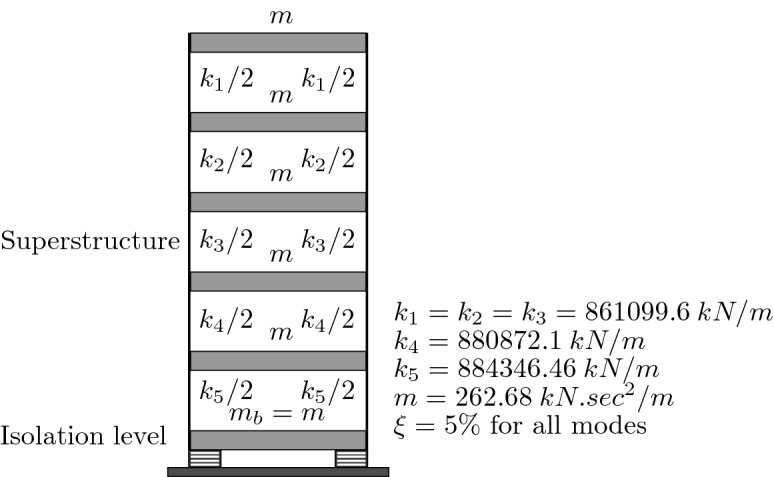
Five-story RC base isolated building.

The analysis procedure followed in the present parametric study is described below:

a.: *Structural model*: Figure 10 illustrates the structure chosen for the parametric analysis analyzed; it is a five-story reinforced concrete building with total mass equal to 1313.38 kN.s$^2$/m uniformly distributed over floors. The fundamental period of the fixed base building modeled as shear structure $T_s = 0.38$ s. The seismic isolation devices are mounted at a base level having the same floor mass, under every column of the base.
b.: *Response parameters of interest*: The response parameters of interest that are considered to be indicators of effectiveness of a seismic isolation are: the peak base displacement, peak structural acceleration, and maximum base shear. Other parameters are will also be briefly analyzed.
c.: *Nonlinear dynamic time history analysis*: High fidelity analyses and extensive evaluation of the influence of selected parameters were deemed paramount for a successful investigation. To that end, the developed numerical integration code was run with a fixed time step length of 0.005 s for the linear anad nonlinear analyses.
d.: *Basic approach to analysis*: First, the structure is studied as fixed at its base then adding an isolation system with different parameters and perform a nonlinear time history analysis for the base isolated structure and a linear time history analysis for the fixed base structure, assuming eight earthquakes. The total number of analyses performed for both structures throughout the study is 11+288=299 time history analyses.
e.: *Seismic isolation design parameters*: The seismic isolation design parameters are: (1) the degree of nonlinearity $1/\alpha$, $\alpha$ is defined as the postyield ($k_p$) to preyield stiffness ($k_e$) ratio and (2) the yield strength level $\mu$, defined as the ratio of yield strength (*Q*) to the total weight of the structure including the base (*W*).
f.: *Input Ground Motions*: The eight earthquake records used in this study are the strong components of the suite of earthquakes specifically suggested by the CDMG (California Division of Mines and Geology, Sacramento, USA) for the design of seismic isolated structures^5^.

### Parametric study considerations

The main aim of the parametric study is to identify the near-optimal seismic isolation design parameters. The behavior of a base isolated buildings subjected to earthquake ground motions is affected by a number of variables mostly related to the isolation system parameters and the characteristics of the ground motion. Jangid^30^ concluded from his parametric study that the damping and period of the superstructure do not have noticeable effects on the peak response base isolated structure. Therefore, in the present study, the superstructure’s parameters were held fixed while the variables that are expected to have a significant effect on the response of the structure were varied. The postyield stiffness $k_p$ of the isolation system was fixed so as to achieve an isolation period, $T_b=2.5$ s:

$$\begin{aligned} k_p=4\pi ^2/T_{b}^{2}(5m+m_b)=4\pi ^2/(2.5)^2(6\times 262.68)=9955.28\;\text{ kN/m. } \end{aligned}$$

**Figure 11:**
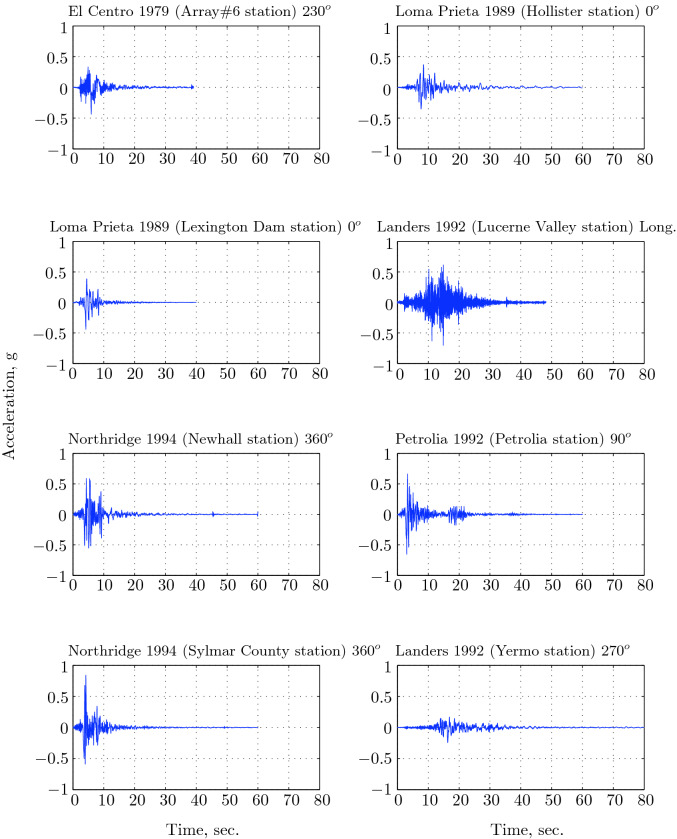
Acceleration time histories of CDMG suite of earthquakes.

**Figure 12:**
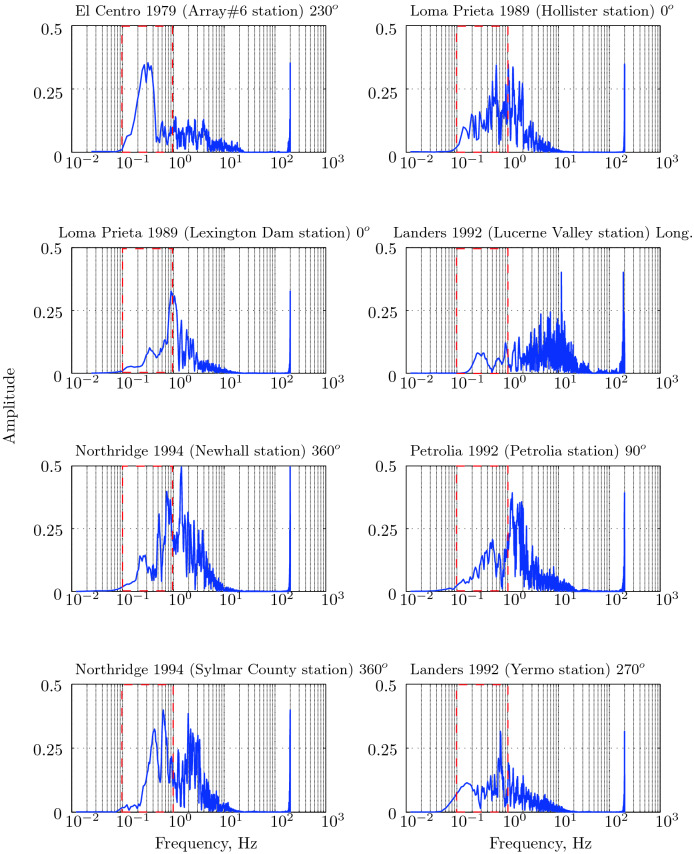
Fourier spectra for CDMG suite of earthquakes.

**Figure 13:**
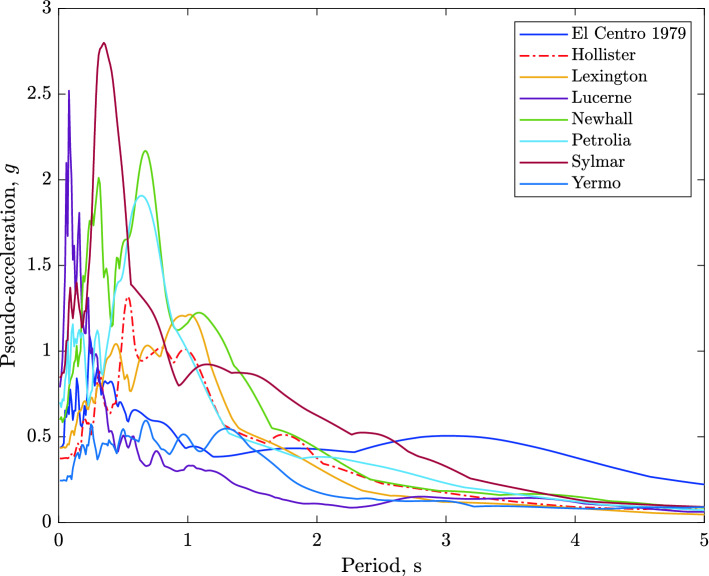
Pseudo-acceleration response spectra for the CDMG suite of earthquakes, $\zeta =5\%$.

The eight earthquake records used in this study are those specifically suggested by the CDMG for design of seismic isolated structures. Table 1 reports the strong component of each earthquake ground motion considered with their parameters. The ground acceleration time histories , their Fourier spectra, and pseudo-acceleration response spectra are shown in Figs. 11, 12, and 13.

**Table 1:** Strong component of CDMG suite of earthquakes.

Earthquake	Station	Component	PGA (*g*)	PGV (cm/s)	PGD (cm)
El Centro 1979	Array#6	$230^o$	0.436	108.709	55.165
Loma Prieta 1989	Hollister	$0^o$	0.369	62.779	30.176
Loma Prieta 1989	Lexington dam	$0^o$	0.442	84.434	14.673
Landers 1992	Lucerne valley	Long.	0.703	25.718	8.824
Northridge 1994	Newhall	$360^o$	0.589	94.719	30.474
Petrolia 1992	Petrolia	$90^o$	0.662	89.454	30.577
Northridge 1994	Sylmar	$360^o$	0.843	128.884	32.550
Landers 1992	Yermo	$270^o$	0.245	50.812	41.275

As we will see later, the severity of an earthquake for base isolated structures is not related to the peak ground acceleration, PGA, but very much related to the peak ground velocity, PGV, and peak ground displacement, PGD. Since the fundamental frequency of a base isolated structure is located in the region of low frequency (below $1\;Hz$), the ground motions (Fig. 12) with considerable low frequency energy are an extremely severe inputs for the isolation systems. Nonlinear dynamic time history analyses were carried out for each combination design variables. For each ground motion input, the total number of analyses performed is $6\times 6\times 8=288$ nonlinear time history analyses, assuming 1/$\alpha$=5, 7, 10, 12, 15, 20 (6 variations) and $\mu$=4%, 5%, 8%, 10%, 12% and 15% (6 variations). The strategy of parameter variations has its basis; the resulting isolation system parameters are the most reasonable and practical.

The parametric analysis reported in the present work considered the effect of varying the design parameters on the main response parameters of the base-isolated structure such as base shear and effective damping. However, it is important to consider uncertainties inherent in the design parameters themselves. Although this study is limited to the deterministic case discrete variations in design parameters, sophisticated approaches are available when considering Uncertainty Quantification (UQ).

## Results and discussion

Data from dynamic analyses consist of plots of response quantities directly related to the specific objectives of this study. The results and discussions are given for each earthquake input and then an overall analysis for the CDMG suite is given at the end to analyze the effect of ground motion parameters on the peak response of base isolated structures.

### Analysis for individual ground motions

**Figure 14:**
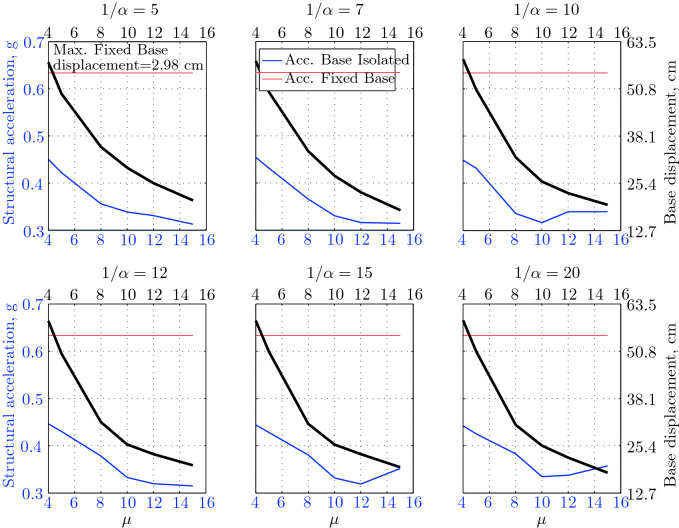
Peak responses of BI-structures subjected to El Centro 1979.

**Figure 15:**
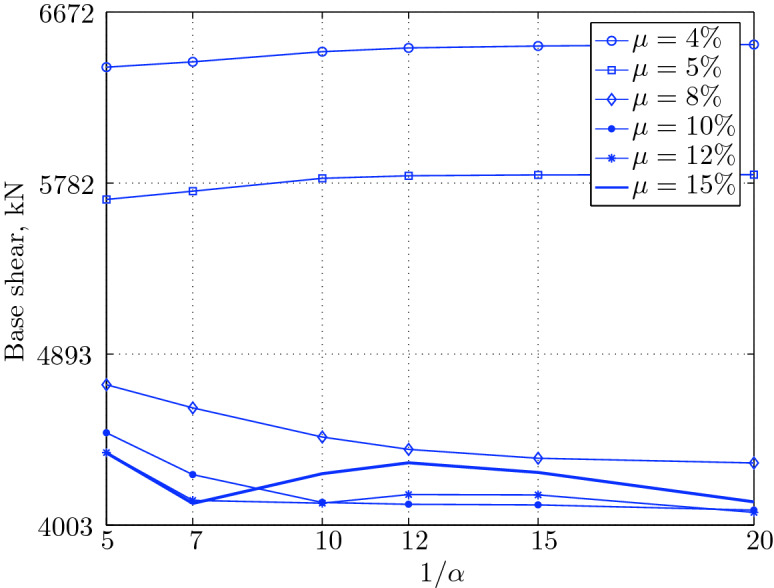
Maximum base shear for various isolation system parameters (El Centro 1979).

**Figure 16:**
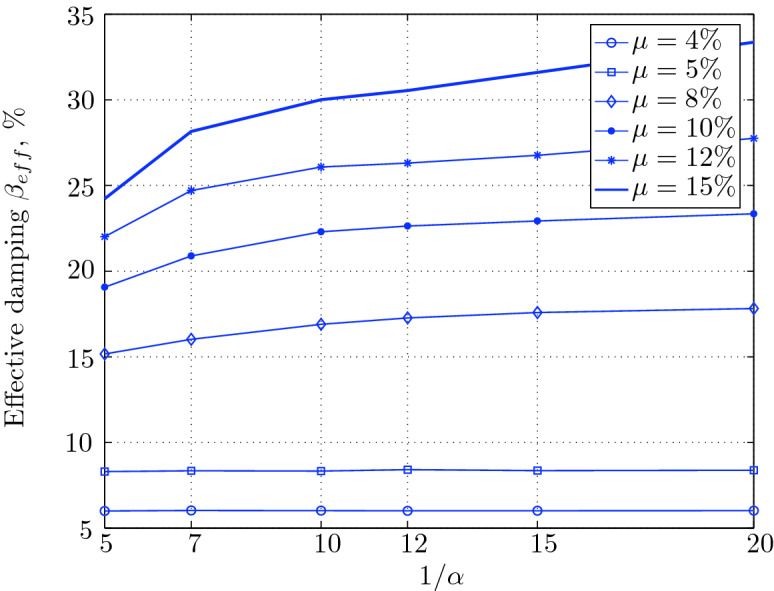
Effective damping for various isolation system parameters (El Centro 1979).

**Figure 17:**
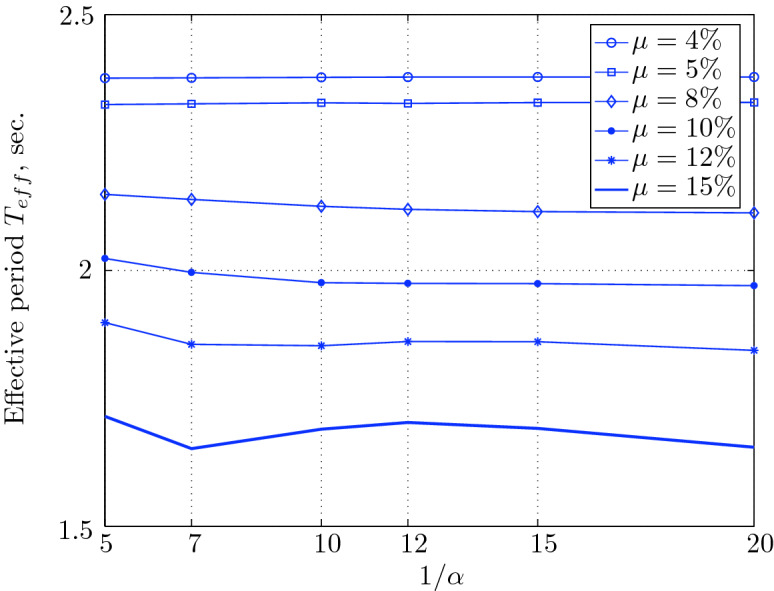
Effective period for various isolation system parameters (El Centro 1979).

*El Centro 1979 (Array#6 station)* $230^o$ The maximum structural acceleration (roof acceleration) and maximum base displacement of base isolated structures subjected to the El Centro 1979 $230^o$ are shown in Fig. 14 (double axis plots) for fixed values of $1/\alpha$. It can be seen from these plots that for low yield strength levels ($\mu =4$% to 6%), the degree of nonlinearity, $1/\alpha$ and hence the preyield stiffness $k_e$, has no noticeable effect on maxima of base displacement and structural acceleration, in this case, the response is controlled mostly by the yield strength level. Figure 15 which shows the plots of maximum base shear with variation of base isolation system parameters, prove the above interpretation. The reduction in maximum base shear observed when increasing $\mu$ from 4 to 5% (but we still in the same region of low yield strength levels) is due mainly to the amount of extra damping, this is well seen in Fig. 16, even the effective period is slightly reduced (see Fig. 17).

Contrarily, at high yield strength levels (from $\mu =8$% to 15%), the degree of nonlinearity, and hence the preyield stiffness controls the maximum base shear, and no noticeable effect of $\mu$ in this range is observed. In addition, the maximum acceleration and interstory drift are slightly constant in this case. The situation is slightly different for maximum base displacement, where the yield strength level continues also to control. Furthermore, in this case, the increase in effective damping has no significant influence in reducing the base shear and structural acceleration even it reaches values higher than 20%.

It is clear from these figures that, even the preyield stiffness controls the peak response in the region of high level of yield strength, a small change in 1/$\alpha$ does not change dramatically the results, and it changes them with very small amounts. So, it is evident that a value of $\mu$ in this case and any value of $1/\alpha$ give approximately the same magnitude of peak response.

The optimal seismic isolation design parameters are normally those with when the base displacement, structural acceleration and base shear are found to be the smallest. Based on this criteria and Figs. 14, 15, 16, 17, it can easily realized that the region of optimal design parameters is located exactly between $\mu =10$% and 15% with any value of preyield stiffness (or $1/\alpha$), since it does not dramatically change the results.

A value of $\mu =15$% and $1/\alpha =7$ are the optimal design parameters, since for this pair we have the smallest structural acceleration, the smallest base displacement, the second smallest base shear and a small interstory drift.

**Figure 18:**
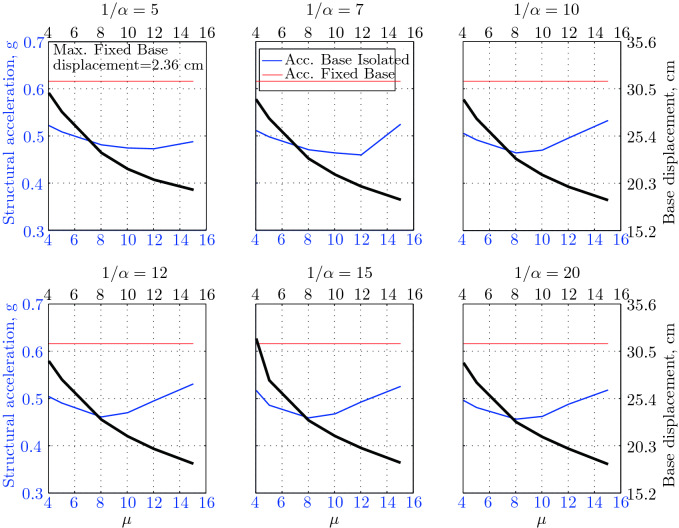
Peak responses of BI-structures subjected to Loma Prieta 1989 (Hollister station).

**Figure 19:**
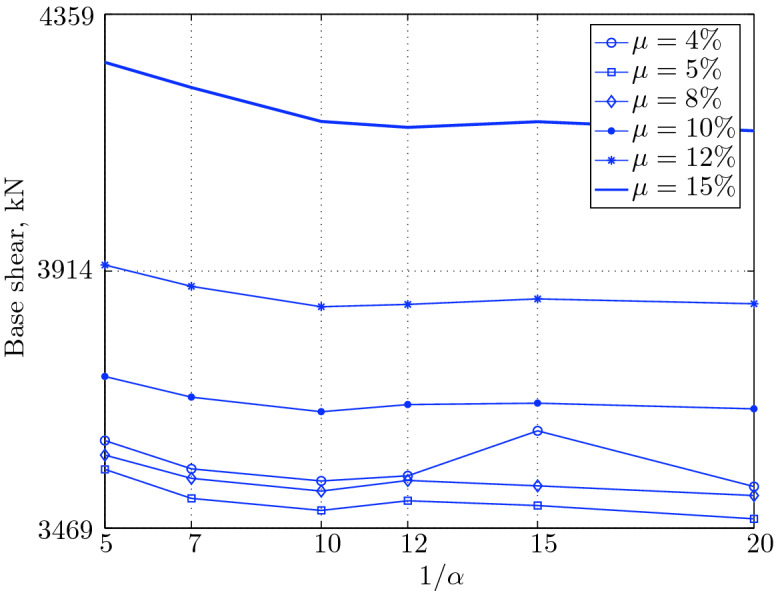
Maximum base shear for various isolation system parameters (Hollister).

**Figure 20:**
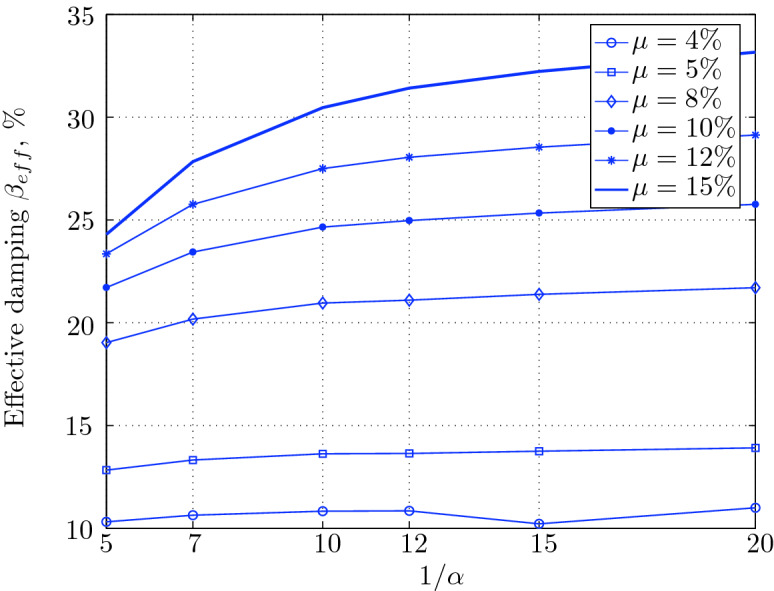
Effective damping for various isolation system parameters (Hollister).

**Figure 21:**
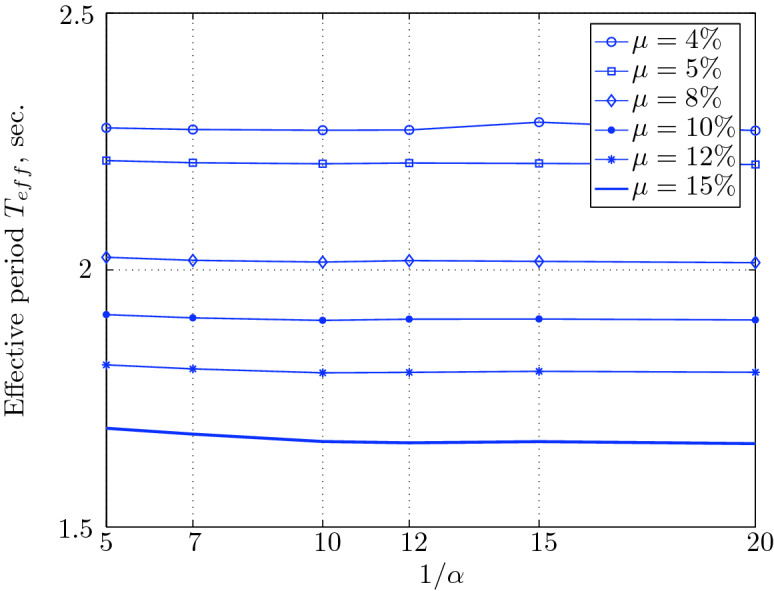
Effective period for various isolation system parameters (Hollister).

*Loma Prieta 1989 (Hollister station)* $0^o$ The maximum structural acceleration (roof acceleration) and maximum base displacement of base isolated structures subjected to the Loma Prieta 1989 earthquake recorded at Hollister station are shown in Fig. 18 for fixed values of $1/\alpha$. Also, is shown in Fig. 19, the maximum base shear for different seismic isolation parameters, and Figs. 20 and 21 show the variation of effective damping and effective period with the same parameters.

Low yield strength levels cause large base displacement (Fig. 18) because of the small amount of effective damping $\beta _{eff}$ (Fig. 20); even the largest effective period $T_{eff}$ (Fig. 21) is located in this region. As the yield strength level increases, maximum base displacement becomes smaller; this is due mainly to the added hysteretic damping.

A base isolation system with large yield strength levels induces greater base shear, great structural acceleration, and small base displacement. The increase in base shear is due mostly to the shorter effective period. In general, for base isolated structures subjected to this earthquake, the influence of the degree of nonlinearity is not significant on the peak response. The yield strength level has a noticeable effect on the maximum base displacement and maximum base shear, but its influence reduces for maximum structural acceleration; since the largest and smallest maximum structural acceleration are close (difference of $0.77\;m/sec^2$).

Based on Figs. 18, 19, 20, 21, it is clear that the region of optimal design parameters is located exactly between $\mu$=6% and 10% with any value of preyield stiffness (or $1/\alpha$), since it has no noticeable influence on the peak response. A value of $\mu$=8% and $1/\alpha$=20 are the optimal design parameters, since for this pair we have the smallest structural acceleration, the smallest interstory drift, a small base shear and a small base displacement.

**Figure 22:**
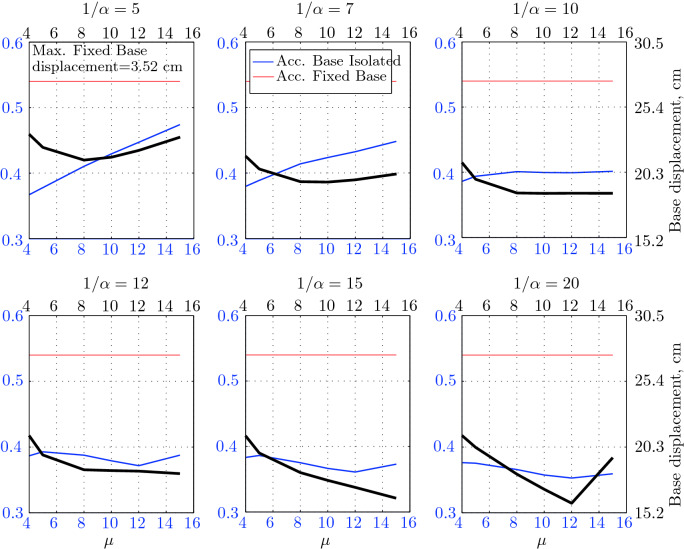
Peak responses of BI-structures subjected to Loma Prieta 1989 (Lexington dam station).

**Figure 23:**
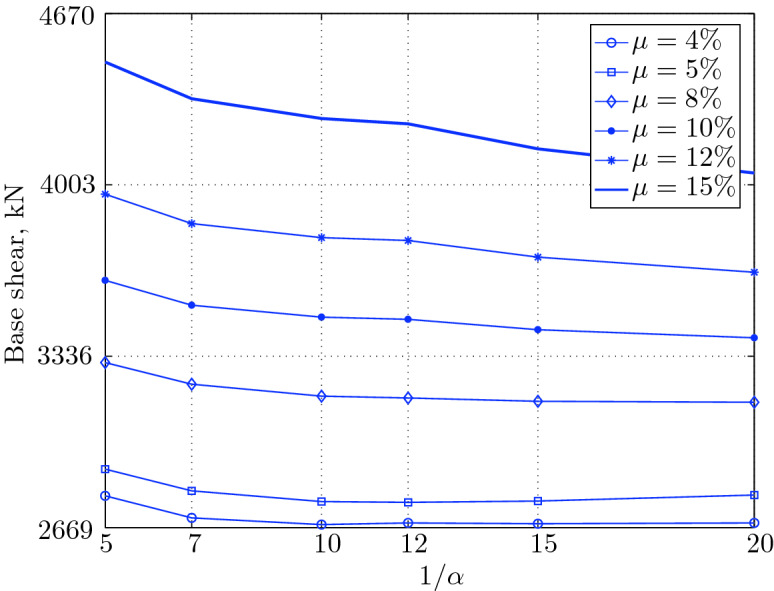
Maximum base shear for various isolation system parameters (Lexington dam).

**Figure 24:**
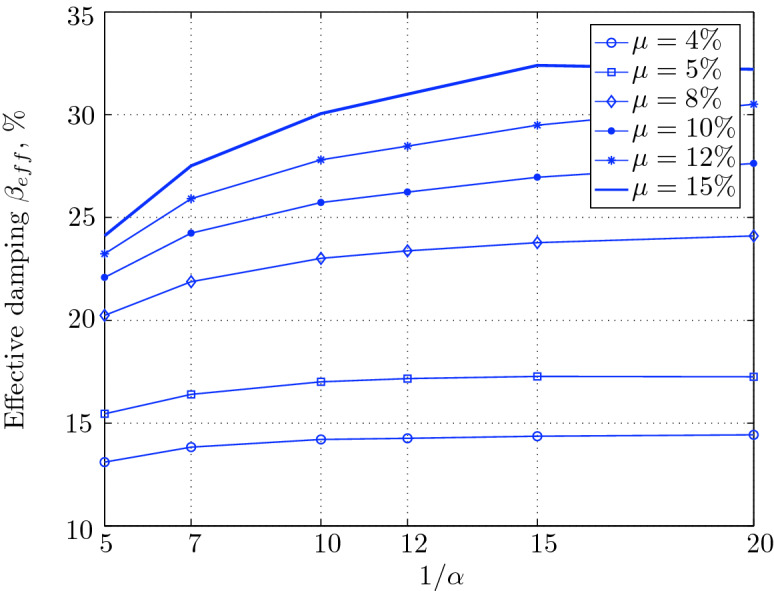
Effective damping for various isolation system parameters (Lexington dam).

**Figure 25:**
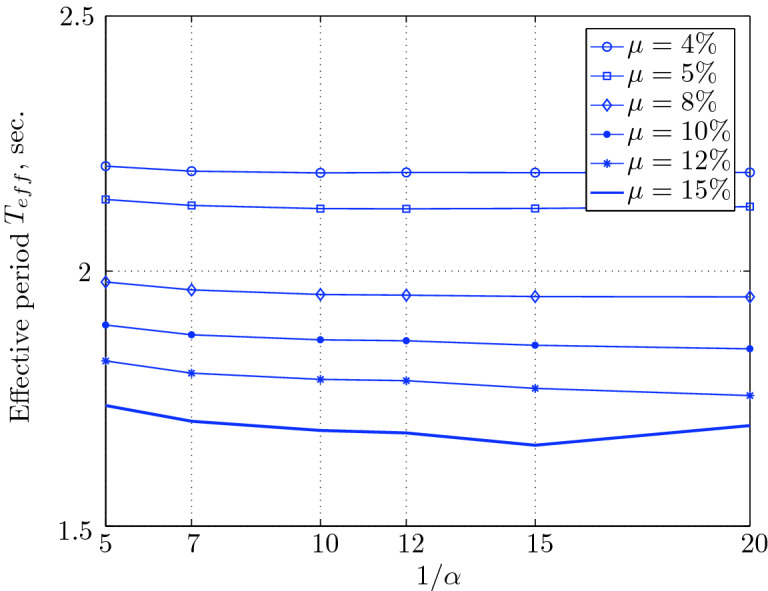
Effective period for various isolation system parameters (Lexington dam).

*Loma Prieta 1989 (Lexington Dam station)* $0^o$ The maximum structural acceleration (roof acceleration) and maximum base displacement of base isolated structures subjected to the Loma Prieta 1989 earthquake recorded at Lexington Dam station are shown in Fig. 22 for fixed values of $1/\alpha$. Also, is shown in Fig. 23, the maximum base shear for different seismic isolation parameters, and Figs. 24 and 25 show the variation of effective damping and effective period with the same parameters.

At low yield strength levels, the degree of nonlinearity, and hence the preyield stiffness has no noticeable effect on the peak response in general (Figs. 22 and 23), but has a small effect on the maximum structural acceleration and the maximum displacement (Fig. 22).

As the yield strength level increases as the maximum base shear, and the preyield stiffness (or $1/\alpha$) begins to influence (Fig. 23). Therefore, at large yield strength levels, the preyield stiffness controls but slightly the motion, in conjunction with the yield strength level, in this case, the base shear becomes larger and base displacement reduces but with small amount while comparing with the largest value observed at lowest yield strength level.

For yield strength levels between $\mu$ = 4 and 5%, the effective stiffness is somewhat constant even the preyield stiffness reaches its largest value, also, the effective damping (Figs. 24 and 25), hence, a smallest energy dissipation capacity. In this case, the peak structural acceleration is influenced mainly by the yield strength level. The significant effect of the preyield stiffness is observed in the region of high yield strength levels ($\mu$ = 8 to 15%), where the two parameters influence the peak response, but not as dramatically as the increase in the maximum base shear. In this region, the preyield stiffness reduces the structural acceleration.

The effective damping effect in general is reduced when large yield strength is given to the seismic isolation system, even the capacity to dissipate energy is higher in this case; this is due mainly to the shorter period associated to system.

Based on Figs. 22, 23, 24, 25, it is clear that the region of optimal design parameters is located exactly between $\mu$=4% and 5% with any value of preyield stiffness (or $1/\alpha$), since it has no great influence on the peak response in this region.

A value of $\mu$=4% and $1/\alpha$=20 are the optimal design parameters, since for this pair we have one of the smallest values of maximum base shear, one of the smallest interstory drift, a small structural acceleration and a small base displacement.

**Figure 26:**
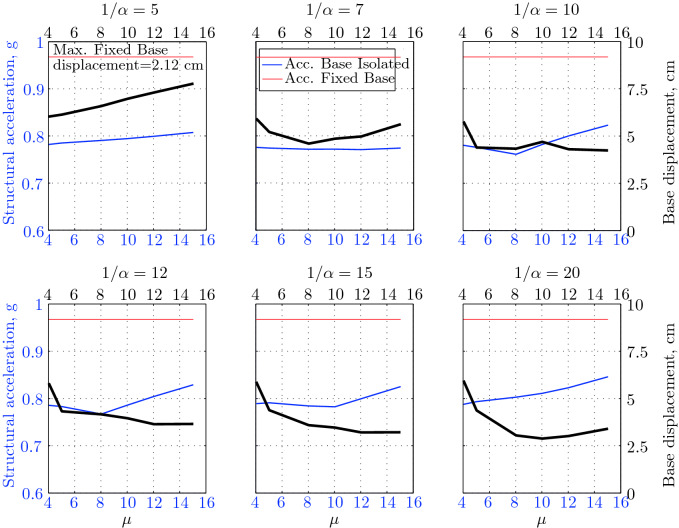
Peak responses of BI-structures subjected to Landers 1992 (Lucerne station).

**Figure 27:**
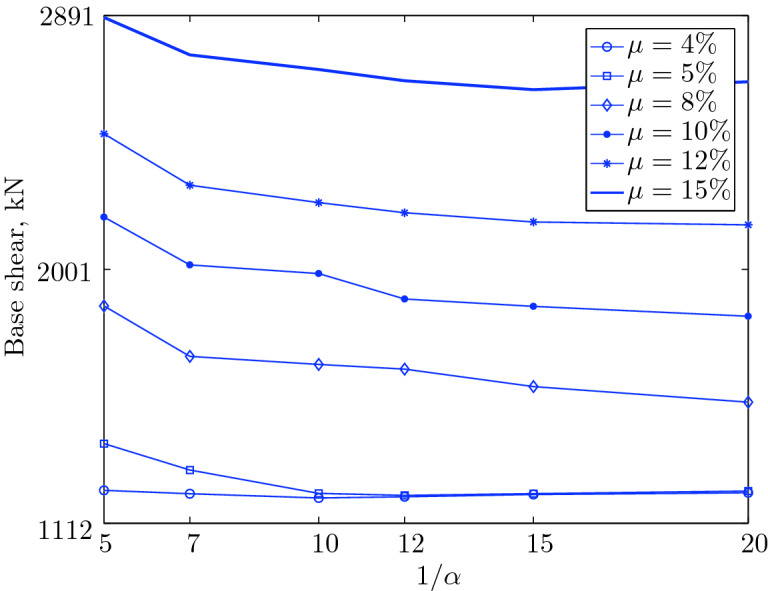
Maximum base shear for various isolation system parameters (Lucerne).

**Figure 28:**
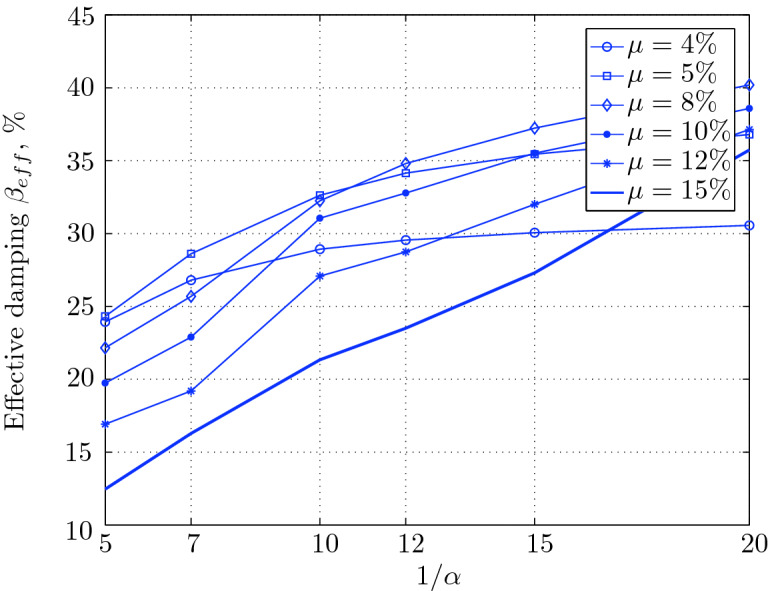
Effective damping for various isolation system parameters (Lucerne).

**Figure 29:**
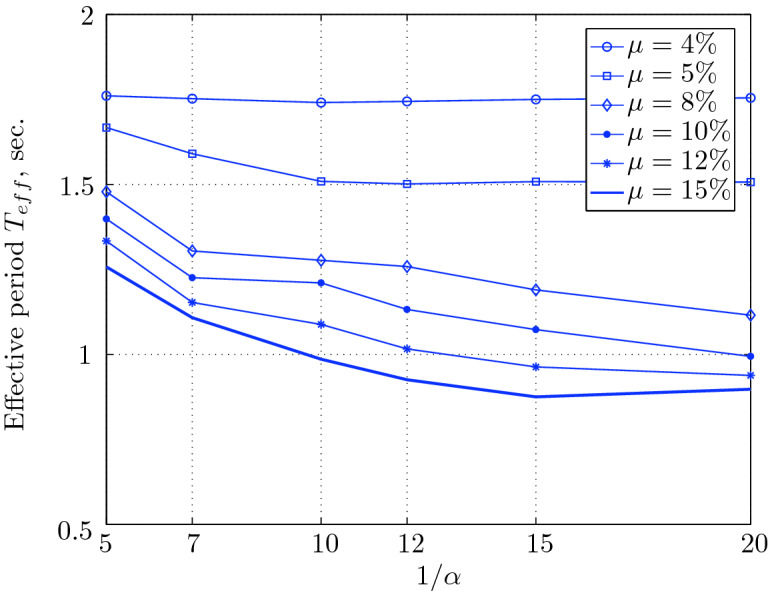
Effective period for various isolation system parameters (Lucerne).

*Landers 1992 (Lucerne valley station) Long.* The maximum structural acceleration and maximum base displacement of base isolated structures subjected to the Landers 1992 earthquake recorded at Lucerne Valley Station (longitudinal component) are shown in Fig. 26 for fixed values of $1/\alpha$. Also, is shown in Fig. 27, the maximum base shear for different seismic isolation parameters, and Figs. 28 and 29 show the variation of effective damping and effective period with the same parameters.

The maximum base displacement observed in Fig. 26 is $7.89\;cm$, which is a very small displacement, regarding the other values observed for the remainder of suite of earthquakes; this can be interpreted by the small peak ground displacement, which is equal to $8.82\;cm$.

At low yield strength levels, large preyield stiffness and hence high degree of nonlinearity doesn’t contribute noticeably on the maximum base shear (Fig. 27) and maximum structural acceleration (Fig. 26). This is due to the fact that the effective period and energy dissipation capacity of the structure do not change in this range ($\mu$=4% to 5%). Also, in this case ($\mu$=4% to 5%) the maximum base is constant and no effect of $1/\alpha$ is observed.

In contrast, at high yield strength levels ($\mu \ge$8%), the effect of the degree of nonlinearity become noticeable in reducing the base displacement, because of the induced extra amount of effective damping (Fig. 28) and hence the capacity to dissipate more ground motion energy. In addition to the significant effect of yield strength level on the peak structural acceleration, the degree of nonlinearity (i.e., the preyield stiffness) also influences, so that for high degree of nonlinearity, the isolation system induces an extra acceleration to the superstructure, but stills small (Fig. 26). However, the maximum base shear is mostly governed by the yield strength and little by the preyield stiffness.

Based on Figs. 26, 27, 28, 29, it is clear that the region of optimal design parameters is located between $\mu$= [4%, 5%] and $1/\alpha$ = [10,20]. A value of $\mu$ = 4% and $1/\alpha$ = 10 are the optimal design parameters, since for this pair we have the smallest maximum base shear, the smallest interstory drift, a small structural acceleration, but this pair does not give a small base displacement comparing with other pairs. However, the base displacement associated to this pair is $6.17\;cm$, which stills small enough if the problem of pounding is under consideration also. So, in the case of small displacements, one have to be aware about the base shear and structural accelerations, those, are the better indicators of effectiveness of such a seismic isolation system.

**Figure 30:**
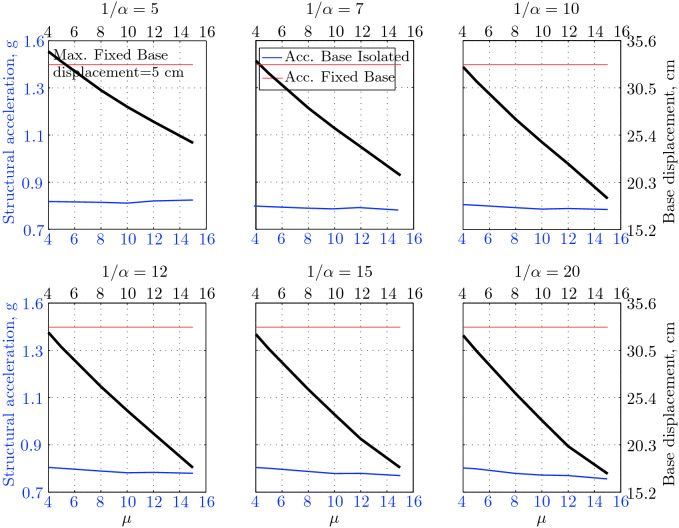
Peak responses of BI-structures subjected Northridge 1994 (Newhall station).

**Figure 31:**
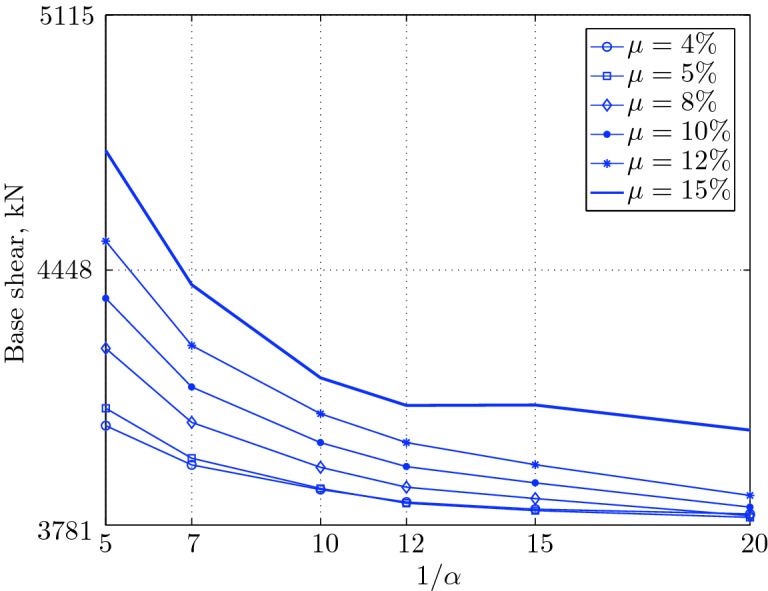
Maximum base shear for various isolation system parameters (Newhall).

**Figure 32:**
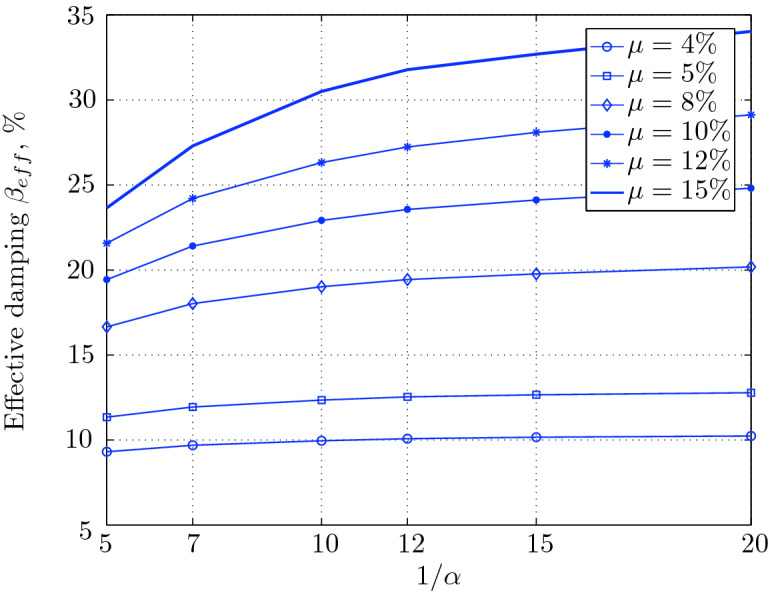
Effective damping for various isolation system parameters (Newhall).

**Figure 33:**
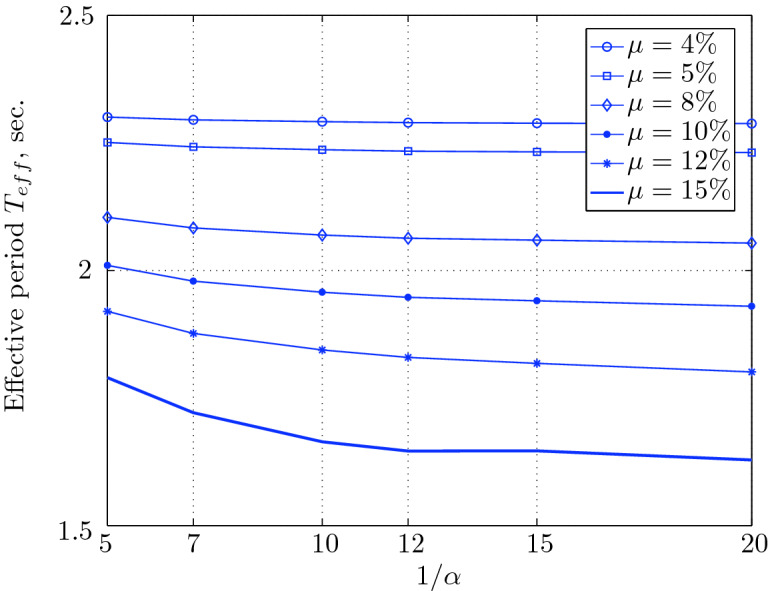
Effective period for various isolation system parameters (Newhall).

*Northridge 1994 (Newhall station)* $360^o$ The maximum structural acceleration and maximum base displacement of base isolated structures subjected to the Northridge 1994 earthquake recorded at the Newhall station $360^o$ are shown in Fig. 30 for fixed values of $1/\alpha$. Also, is shown in Fig. 31, the maximum base shear for different seismic isolation parameters, and Figs. 32 and 33 show the variation of effective damping and effective period with the same parameters.

At low levels of yield strength ($\mu$=4%-5%), the maximum base shear (Fig. 31) is governed mostly by the degree of nonlinearity (i.e., preyield stiffness) in this case, a high degree of nonlinearity reduces the maximum base shear, but its effect tends to be not significant for higher ranges; when the preyield stiffness becomes more than 10 times the postyield stiffness. However, in this case (low yield strength levels), the degree of nonlinearity does no more govern the maximum base displacement (Fig. 30); rather, it is the yield strength in this case that governs this response quantity. Furthermore, that is not the case for structural acceleration (Fig. 30) which is governed strictly by the degree of nonlinearity, when the preyield stiffness is less than 7 times the postyield stiffness, when this ratio becomes larger, which means high degree of nonlinearity, the yield strength begins to govern also the motion, but for higher degree of nonlinearity, the peak structural acceleration becomes strictly controlled by the yield strength level.

At high levels of yield strength ($\mu \ge$8%), the latter influences noticeably the maximum base shear and maximum base displacement in conjunction with the degree of nonlinearity. However, the influence of the preyield stiffness becomes insignificant when it is larger than 10 times the postyield stiffness, this can be explained by the fact that small extra amount of effective damping is added (Fig. 32). At these levels of yield strength ,the structural acceleration is governed mostly by the degree of nonlinearity; a high degree of nonlinearity reduces the peak structural acceleration. The yield strength becomes effective just for seismic isolation systems with very high degree of nonlinearity, say more than 15%.

For this earthquake, the situation is quite difficult to pick the region of optimal design parameters that lead to best performance; because the smallest maximum structural acceleration and base displacement are reached for seismic isolation system having high yield strength level ($\mu$=15%) and high degree of nonlinearity ($1/\alpha$=20), but the maximum base shear is reached when the isolation system has a low yield strength level ($\mu$=5%) and a high degree of nonlinearity ($1/\alpha$=20). However, based on Figs. 30 to 33, the region of optimal design parameters can be located somewhere between $\mu$=5% and 10% with the highest degree of nonlinearity, $1/\alpha$=20. However, the pair ($\mu$=8%, $1/\alpha$=20) can be considered as the optimal design parameters.

**Figure 34:**
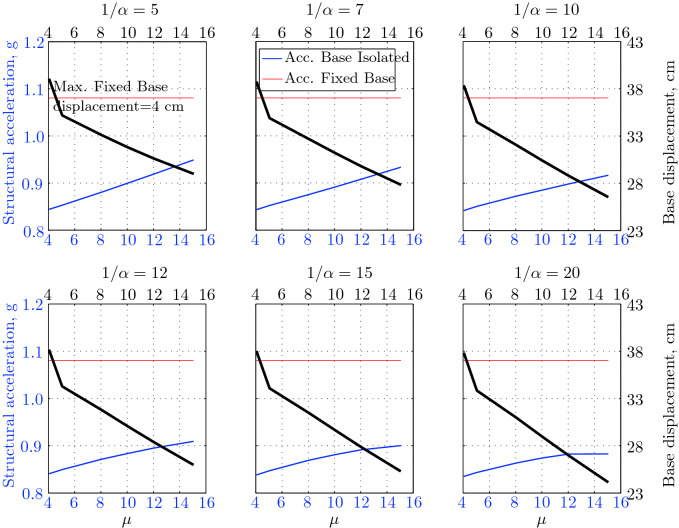
Peak responses of BI-structures subjected to Petrolia 1992 (Petrolia station).

**Figure 35:**
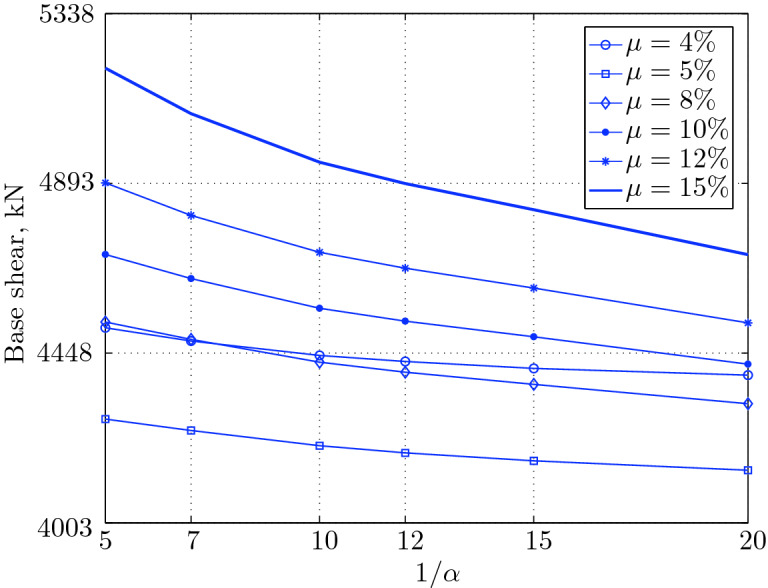
Maximum base shear for various isolation system parameters (Petrolia).

**Figure 36:**
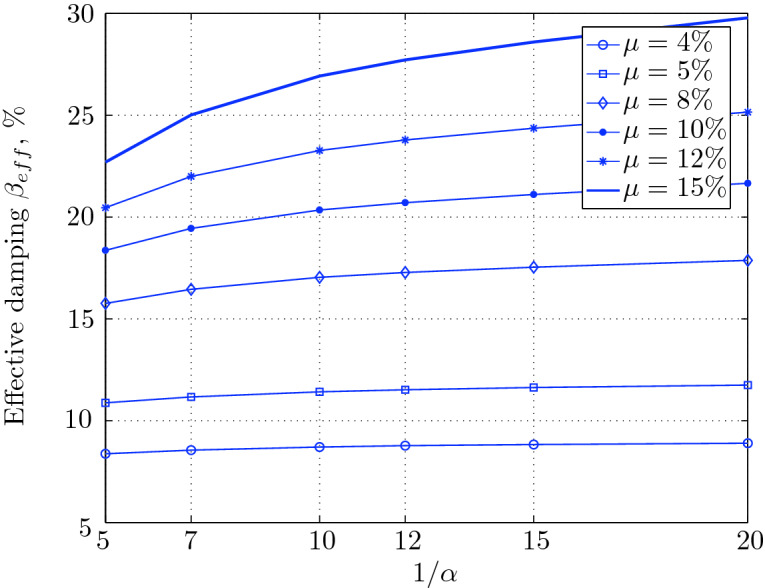
Effective damping for various isolation system parameters (Petrolia).

**Figure 37:**
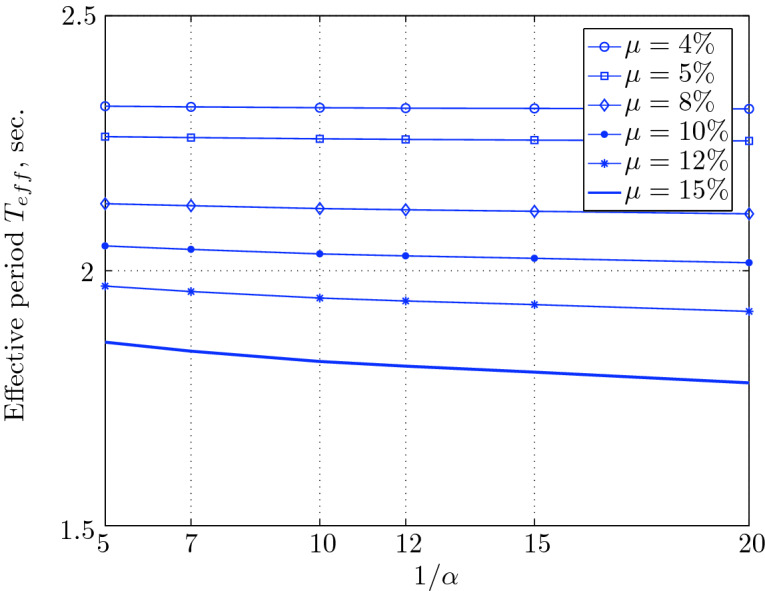
Effective period for various isolation system parameters (Petrolia).

**Petrolia 1992 (Petrolia station)** $90^o$ The maximum structural acceleration and maximum base displacement of base isolated structures subjected to the Petrolia 1992 earthquake recorded at the Petrolia station $90^o$ are shown in Fig. 34 for fixed values of $1/\alpha$. Also, is shown in Fig. 35, the maximum base shear for different seismic isolation parameters, and Figs. 36 and 37 show the variation of effective damping and effective period with the same parameters.

As seen from Figs. 34 and 35, it is clear that the peak response is governed by the yield strength level, and the degree of nonlinearity begins to contribute in governing the peak response at the region of high yield strength levels. The maximum base shear tends to be smaller as the yield strength level reaches 5%, after that the maximum becomes larger as the yield strength level. The maximum structural acceleration follows in this case the yield strength levels. In contrast, the maximum displacement becomes smaller as the yield strength reaches its largest value ($\mu$=15%) at higher degree of nonlinearity.

To select the region of optimal design parameters, one must decide what is the main response quantity that will dominate the choice of the base isolation system to be used? If the base displacement is very important, when the problem of pounding is present, we have to choose isolation system with high yield strength and high degree of nonlinearity; in this case we will lose the benefits of more reducing the base shear and structural acceleration. Contrarily, if the above problem is not important, one can gain more benefits of reducing the structural acceleration and base shear at the same time with the same parameters but with larger base displacement; in this case choosing an isolation system with low yield strength level with high degree of nonlinearity is the best choice.

However, in our case, a yield strength level between 4% and 10% with any degree of nonlinearity (or $1/\alpha$=15−20) is the near optimal choice. The pair ($\mu$=5%, $1/\alpha$=20) can be considered as the optimal design parameters, since they cause a smallest maximum base shear, a smallest structural acceleration and the smallest interstory drift.

**Figure 38:**
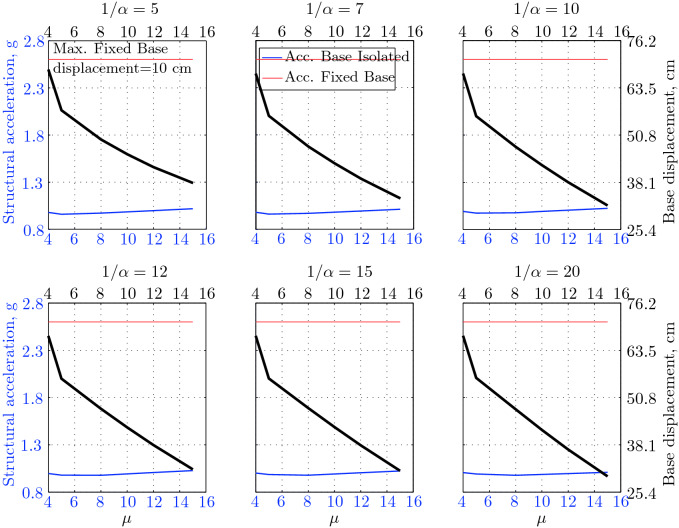
Peak responses of BI-structures subjected to Northridge 1994 (Sylmar station).

**Figure 39:**
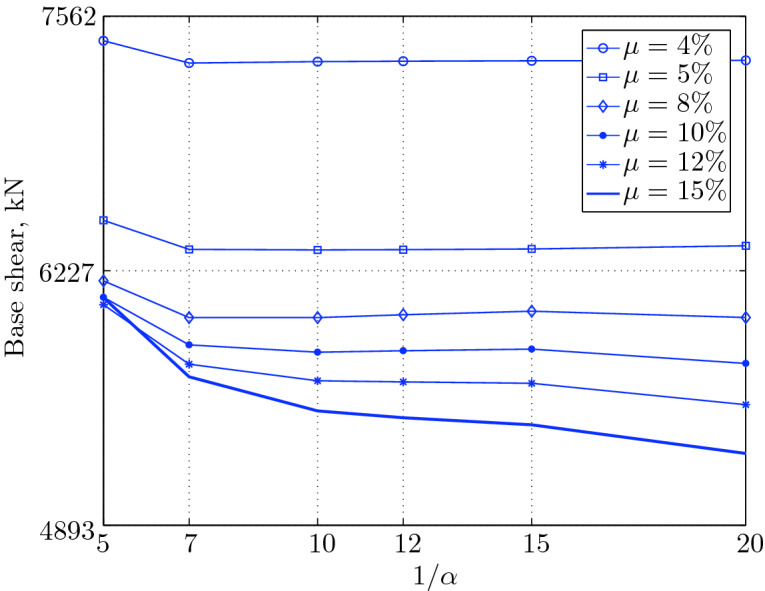
Maximum base shear for various isolation system parameters (Sylmar).

**Figure 40:**
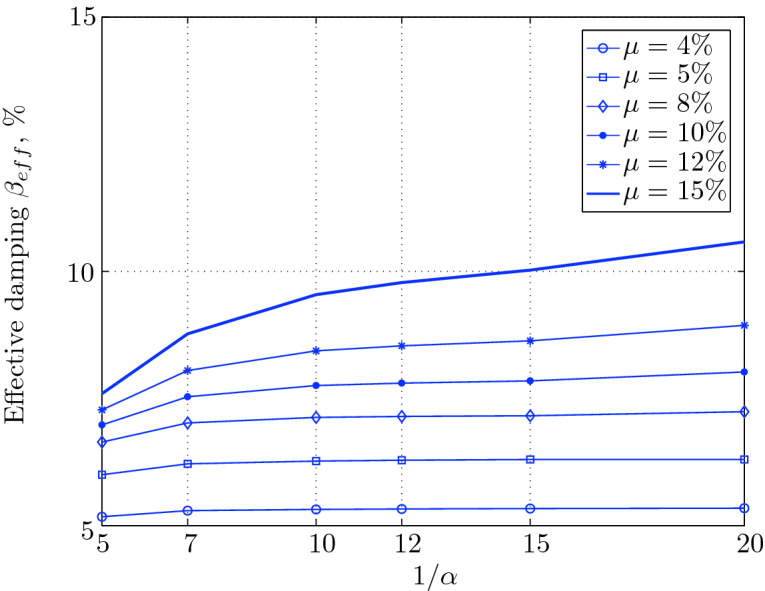
Effective damping for various isolation system parameters (Sylmar).

**Figure 41:**
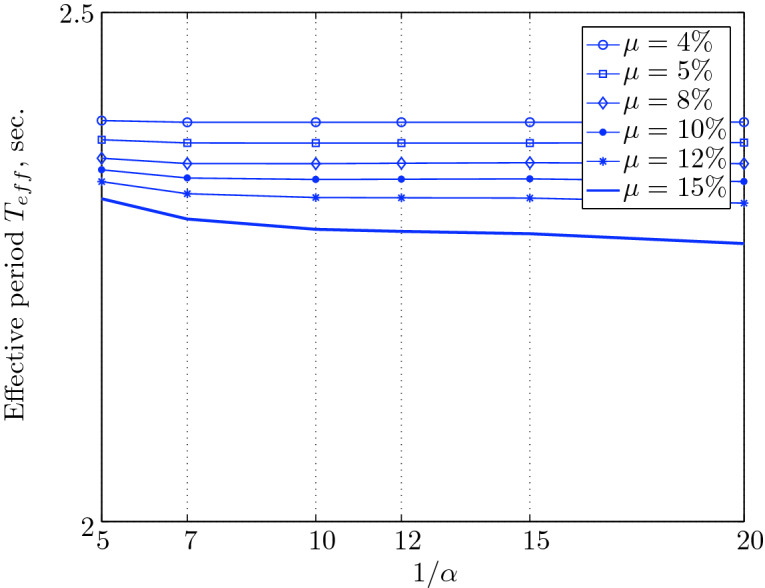
Effective period for various isolation system parameters (Sylmar).

**Northridge 1994 (Sylmar station)** $360^o$ The maximum structural acceleration and maximum base displacement of base isolated structures subjected to the Northridge 1994 earthquake recorded at the Sylmar station $360^o$ are shown in Fig. 38 for fixed values of $1/\alpha$. Also, is shown in Fig. 39, the maximum base shear for different seismic isolation parameters, and Figs. 40 and 41 show the variation of effective damping and effective period with the same parameters.

At low yield strength levels, the degree of nonlinearity has no effect on the peak response of base isolated structures (Figs. 38 and 39); the control in this case belongs to the yield strength level alone. A great decrease of the peak responses is observed from $\mu$=4% and 5%.

At high yield strength levels, the degree of nonlinearity has a negligible effect on the maximum base displacement and maximum structural acceleration. The effect of the degree of nonlinearity is noticeable in the case of case of very high yield strength levels in reducing the maximum base shear. However, in general, the peak response of base isolated structures subjected to this particular earthquake is governed mostly by the yield strength level. The effective damping is not as great as when subjected to other earthquakes, in this case the maximum effective damping is around 10%, which is small.

The capacity to dissipate more energy is very great in this case; regarding that the energy dissipated per cycle is around $2824.25\;kN.m$, that is a great while comparing to the same structures subjected to other earthquakes. A severe earthquake having the character of Northridge 1994 (recorded at Sylmar County) causes the structure to dissipate more energy, so that it can reduce the maximum base shear to 15% of the corresponding fixed base structure. This level of reducing the base shear is the best reduction observed for the entire CDMG suite of earthquakes.

The region of optimal seismic isolation parameters can be selected simply in this case, we can arrive at the smallest maximum base shear with the smallest base displacement, with the pair of isolation system parameters ranging from: $\mu$=10%−15% and a very high degree of nonlinearity, say $1/\alpha$=15−20. The peak structural acceleration varies slightly in this range. The pair [$\mu$=15%, $1/\alpha$=20] gives the smallest maximum base shear, which constitutes 15% of the maximum base shear of the corresponding fixed base structure, and the smallest maximum base displacement, also, this pair gives the smallest interstory drift.

**Figure 42:**
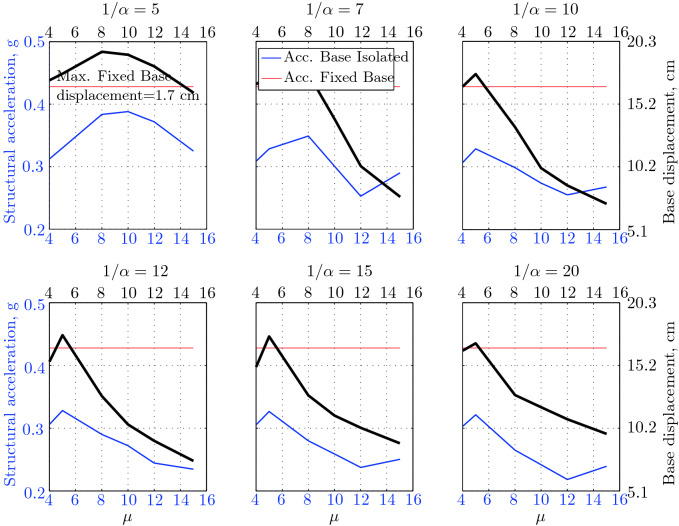
Peak responses of BI-structures subjected to Landers 1992 (Yermo station).

**Figure 43:**
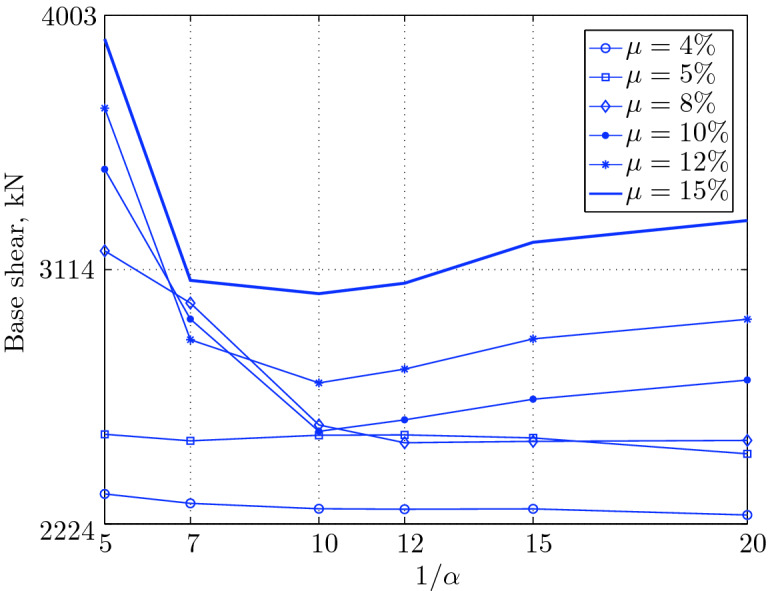
Maximum base shear for various isolation system parameters (Yermo).

**Figure 44:**
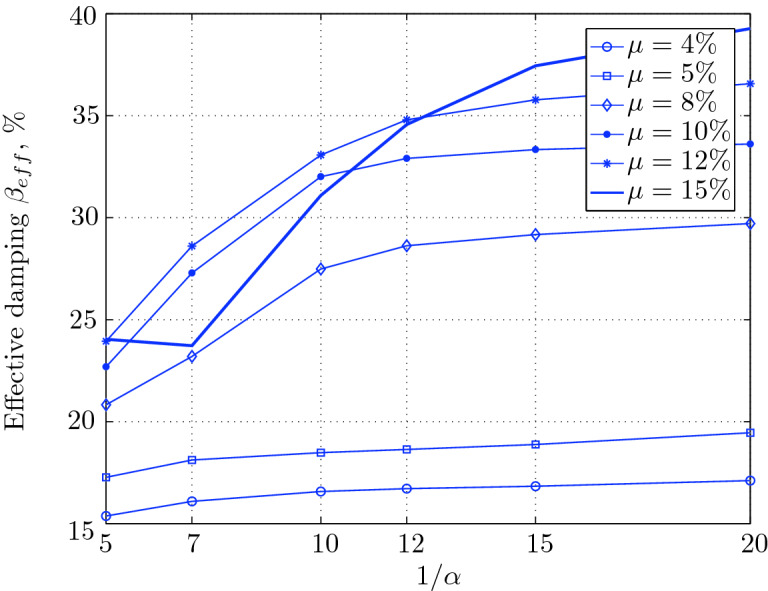
Effective damping for various isolation system parameters (Yermo).

**Figure 45:**
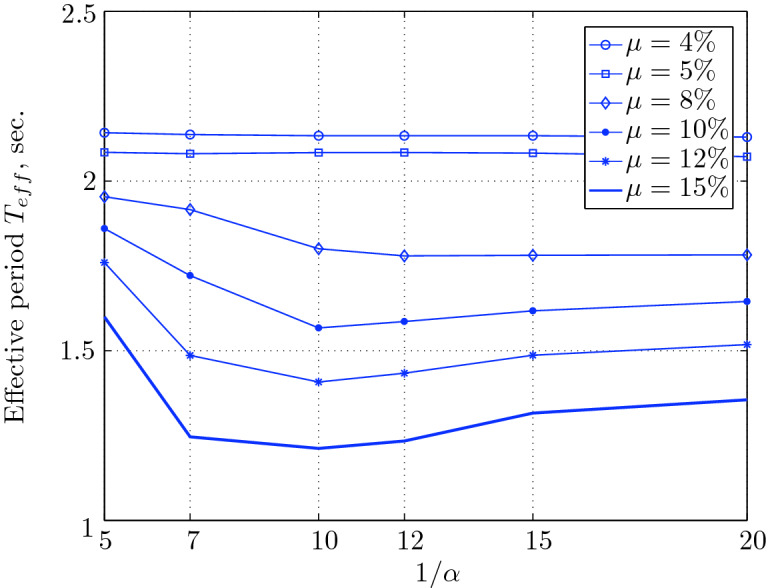
Effective period for various isolation system parameters (Yermo).

**Landers 1992 (Yermo station)** $270^o$ The maximum structural acceleration and maximum base displacement of base isolated structures subjected to the Landers 1992 earthquake recorded at the Yermo station $270^o$ are shown in Fig. 42 for fixed values of $1/\alpha$. Also, is shown in Fig. 43, the maximum base shear for different seismic isolation parameters, and Figs. 44 and 45 show the variation of effective damping and effective period with the same parameters.

At low yield strength levels, low degree of nonlinearity has small effect on the peak response of base isolated structures (Figs. 42 and 43). In this case, the small augmentation in yield strength causes larger maximum base shear, as well as for base displacement and structural acceleration, because the effective period is shortened (Fig. 45), and a very small added effective damping has not effect.

However, an isolation system with high degree of nonlinearity does not affect the peak response, namely the maximum base shear, maximum base displacement, and peak structural acceleration, when high yield strength is used for the isolation system, this is due to the fact the effective damping doesn’t change significantly as well as the effective period, that seem to be constant for a constant high yield strength, and they do not change with the degree of nonlinearity (Figs. 44 and 45).

Because of the small variation of the maximum structural acceleration, which its variation is banded between 0.25*g* and 0.36*g*, and because the larger maximum base displacement is in a reasonable range $19.5\;cm$, the maximum base shear in this case is the main performance indicator of a seismic isolation system.

Based on the above consideration, the region of optimal design parameters may be located between $\mu$=4%−5% and any value of $1/\alpha$, or between $\mu$=4%−8% with a high degree of nonlinearity (say $1/\alpha$=10−20). The pair [$\mu$=4%, $1/\alpha$=20] gives the smallest maximum base shear, which constitutes 37.4% of the maximum base shear of the corresponding fixed base structure and the smallest interstory drift.

### Overall analysis for the CDMG suite of ground motions

Table 2 summarizes the near optimal design seismic isolation parameters (region and discrete values) for each earthquake of the CDMG suite in parallel with their characteristics (units for PGA, PGV and PGD are *cm* and *sec*. and the $*$ character means any value) obtained from the parametric analysis.

**Table 2:** Near optimal design seismic isolation parameters for the CDMG suite.

Earthquake	PGA	PGV	PGD	Optimal region		Near opt. param.	
				$\mu (\%)$	$1/\alpha$	$\mu (\%)$	$1/\alpha$
El Centro	427.72	108.71	55.16	[10,15]	*	15	7
Hollister	361.99	62.78	30.18	[6,10]	*	8	20
Lexington	433.60	84.43	14.67	[4,5]	*	4	20
Lucerne	689.64	25.72	8.82	[4,5]	[10,20]	4	10
Newhall	577.81	94.72	30.47	[5,10]	20	8	20
Petrolia	649.42	89.45	30.58	[4,10]	[15,20]	5	20
Sylmar	826.98	128.89	32.55	[10,15]	[15,20]	15	20
Yermo	240.34	50.81	41.27	[4,5]/[4,8]	[10,20]	4	20

#### Base shear

Table 3 The table shows the smallest maximum base shear for each earthquake record, as well as the largest maximum base shear and the corresponding pair of isolation system parameters. The PGV controls the maximum base shear in conjunction with the PGD, but the latter has a very small effect. The PGA has no effect on the maximum base shear of base isolated structures, since they have a long period that puts them in the velocity sensitivity (for those having long isolation period) and in the displacement sensitivity (for those having very long period), also, this is clear from the table. The maximum base shear is reduced slightly for the Newhall earthquake (comparing with Petrolia) even it has greater PGV than Petrolia, this cannot be interpreted by the reduction in PGA, because it has no effect (see for example Lucerne and Yermo in the table), also cannot be interpreted by the PGD, because the reduction in this case is negligible. This can be interpreted by the frequency content.

**Table 3:** Isolation system parameters that give smallest max. base shear.

Earthquake	PGA	PGV	PGD	Min $V_{b\;max}$	$\mu (\%)$	$1/\alpha$	Max $V_{b\;max}$	$\mu (\%)$	$1/\alpha$
Lucerne	689.64	25.72	8.82	685.67	4	10	648.46	15	5
Yermo	240.34	50.81	41.27	1287.86	4	20	881.18	15	5
Hollister	361.99	62.78	30.18	1990.35	5	20	961.25	15	5
Lexington	433.60	84.43	14.67	1530.40	4	10	1007.60	15	5
Petrolia	649.42	89.45	30.58	2364.65	5	20	1167.90	15	5
Newhall	577.81	94.72	30.47	2170.60	5	20	1070.20	15	5
El centro	427.72	108.71	55.16	2323.61	12	20	1462.10	4	20
Sylmar	826.98	128.89	32.55	3008.63	15	20	1671.20	4	5

The base isolated structure (with $\mu$=4% and $1/\alpha =10$) subjected to the Lexington earthquake (PGV = 84.43 cm/sec.) has a maximum base shear smaller than that of the base isolated structure (with $\mu$=5% and $1/\alpha$) when subjected to the Hollister (PGV = 62.78 cm/s); this can be interpreted by the PGD, which is smaller in Lexington . In this case, we can understand why PGA has no effect, because Lexington has PGA greater than Hollister, and the maximum base shear is smaller when the structure is subjected to it.

The CDMG suite of earthquakes can be divided into two main categories; the first category includes El Centro and Sylmar and the second category includes the remainder of earthquakes. The first category has a great PGV, so it includes the severe earthquakes. The second category includes moderate and those having less severity earthquakes.

The largest maximum base shear occurs when using isolation systems with $\mu$=15, that means high yield strength level and $1/\alpha =5$, that means low degree of nonlinearity for the second category. Therefore, one should select an isolation system with low yield strength level (say 4–5%) with high degree of nonlinearity (say $1/\alpha =10-20$ or even more) in order to get smallest maximum base shear. In contrast, a base isolated structure subjected to an earthquake from the second category has a large maximum base shear when the isolation system is characterized by low yield strength level ($\mu =4\%$) and very low degree of nonlinearity. Therefore, the appropriate isolation system must have a high degree of nonlinearity as well as a high yield strength level, in order to meet the performance expected from the seismic isolation system.

#### Base displacement

Table 4 reports the isolation system parameters that give the smallest maximum base displacement and the corresponding maximum base displacement for each earthquake record, also is reported in the same table the largest maximum base displacement and the corresponding pair of isolation system parameters.

**Table 4:** Isolation system parameters that give smallest max. base displacement.

Earthquake	PGA	PGV	PGD	Min $x_{b\;max}$	$\mu (\%)$	$1/\alpha$	Max $x_{b\;max}$	$\mu (\%)$	$1/\alpha$
Lucerne	689.64	25.72	8.82	2.92	10	20	3.11	15	5
Yermo	240.34	50.81	41.27	7.16	15	10	7.68	8	5
Hollister	361.99	62.78	30.18	18.45	15	20	12.58	4	15
Lexington	433.60	84.43	14.67	18.15	12	20	8.59	4	5
Petrolia	649.42	89.45	30.58	23.95	15	20	15.40	4	5
Newhall	577.81	94.72	30.47	17.17	15	20	13.53	4	5
El Centro	427.72	108.71	55.16	18.05	15	7	23.27	4	20
Sylmar	826.98	128.89	32.55	29.62	15	20	26.95	4	5

Tables 2 and 3 show that the maximum base displacement is velocity sensitive, meaning that the PGV governs the motion. The influence of PGD is not as noticeable; it affects the response when the PGV is moderate. There is a trade-off between reducing the base shear and increasing the base displacement, which can be overcome by choosing parameters according to the primary need. In our study, we selected parameters that are near optimal and were chosen to give the best performance expected from the use of an isolation system. Generally, the base shear is considered as the main parameter of interest when selecting the appropriate isolation system parameters, as well as the structural acceleration, which can be amplified (higher modes effect). The base displacement is less important, since the interstory drift is related to base shear.

## Conclusions

Base isolation is a technique used to prevent earthquake-induced damage to a building. Isolation devices are used to decouple the building from the ground, preventing the transmission of earthquake forces to the building. By doing so, the building is able to move more freely, minimizing damage. There are a variety of isolation devices available, each with its own benefits and drawbacks. The selection of an appropriate device is critical to the success of a base isolation system.

In the present study, nonlinear time history analysis and energetic approach were employed to study the advantages and disadvantages of seismic base isolation technology. Base isolation has been demonstrated to be effective in reducing the effects of earthquakes on buildings. However, the selection of the appropriate isolation device is based on a number of requirements which can be complex. This study sought to find the near-optimal design parameters for a base isolated building using a parametric analysis of a number of ground motions. Results showed that the maximum base shear and displacement were velocity-sensitive, and that the PGV controlled the motion. The largest maximum base shear occurred when using isolation systems with high yield strength levels and low degrees of nonlinearity, while the smallest maximum base shear occurred when using low yield strength levels and high degrees of nonlinearity.

This study found that for a fixed value of the degree of nonlinearity, the yield strength has no noticeable effect on the maximum base displacement or structural acceleration for low levels of yield strength ratiod (4–6%). However, for high levels of yield strength ratios (8–15%), the degree of nonlinearity controls the maximum base shear. A base isolation system with large yield strength levels induces greater base shear, great structural acceleration, and small base displacement. The study also found that the region of optimal design parameters is located exactly between $\mu =6\%$ and 10% with any value of preyield stiffness. A value of $\mu =8\%$ and $1/\alpha =20$ are the optimal design parameters. To summarize:

- the base shear is the main parameter of interest when selecting the appropriate isolation system parameters.
- The base displacement is less important, since the interstory drift is related to base shear.
- The PGV governs the motion and the influence of PGD is not as noticeable; it affects the response when the PGV is moderate.
- There is a trade-off between reducing the base shear and increasing the base displacement, which can be overcome by choosing parameters according to the primary need.
- In our study, we selected parameters that are near optimal and were chosen to give the best performance expected from the use of an isolation system.
- Generally, the base shear is considered as the main parameter of interest when selecting the appropriate isolation system parameters, as well as the structural acceleration, which can be amplified (higher modes effect).

## Data Availability

The datasets generated during and/or analysed during the current study are available from the corresponding author on reasonable request.
